# Ligand-Modulated Release of Copper Active Sites Extends Ethylene Production in CO_2_ Electroreduction

**DOI:** 10.1021/jacs.5c22701

**Published:** 2026-03-19

**Authors:** Jari Leemans, Edvin Fako, Ludovic Zaza, Moritz Tritschler, Junwu Chen, Philippe Schwaller, Raffaella Buonsanti

**Affiliations:** † Laboratory of Nanochemistry for Energy, 27218 Institute of Chemical Sciences and Engineering, École Polytechnique Fédérale de Lausanne, 1950 Sion, Switzerland; ‡ Laboratory of Artificial Chemical Intelligence, 27218 Institute of Chemical Sciences and Engineering, École Polytechnique Fédérale de Lausanne, 1015 Lausanne, Switzerland

## Abstract

Copper catalysts for the electrochemical CO_2_ reduction reaction (CO_2_RR) undergo continuous reconstruction, causing a loss of ethylene selectivity over time. Here, we introduce a strategy based on molecular surface chemistry that prolongs the period of maximum ethylene production without sacrificing the activity of Cu electrocatalysts. First, we propose a customized synthesis of colloidal Cu nanocrystals to obtain comparable Cu catalysts differing only in the chemical nature of the organic surface ligand monolayer, thus acting as ideal model systems. Having discovered the unique behavior of secondary phosphines (PR_2_H), we extend the proposed molecular strategy to commercial Cu, achieving a 10-fold increase in the operational stability of ethylene production. Operando spectroscopies combined with ex-situ microscopy correlate slower ligand desorption kinetics to delayed Cu reduction and restructuring, while atomistic simulations indicate that ligands do not intrinsically contribute to ethylene production. Altogether, surface ligands emerge as kinetic gatekeepers that sustain the generation of ethylene-selective active sites by modulating the Cu surface activation. This ligand-modulated activation concept provides a new design principle for possibly improving the long-term stability in electrocatalytic CO_2_RR.

## Graphical abstract

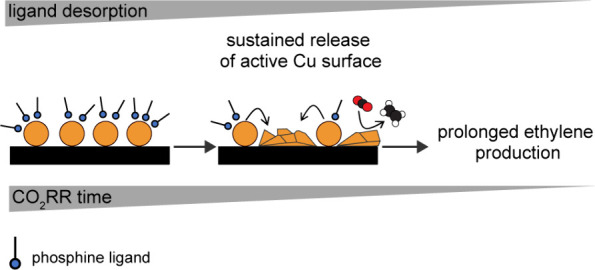

## Introduction

Electrocatalysts deactivate often as a result of structural reconstruction, with involved phenomena spanning from surface atom diffusion and coalescence to dissolution in electrolyte.^1–8^ Copper reconstruction is a prime example and poses one of the main hurdles toward the technological deployment of the electrocatalytic CO_2_ reduction reaction (CO_2_RR).^4^ CO_2_RR is a promising strategy for carbon utilization, forming valuable base chemicals from CO_2_ while using renewable energy.^9^ Thus, CO_2_RR represents a sustainable route to mitigate CO_2_ emissions by valorizing captured CO_2_.

Copper reconstruction during CO_2_RR occurs via several steps, which are well documented in the literature.^10–30^ At start-up, oxidized and dissolved Cu species reduce and redeposit, leading to morphological changes of the Cu catalyst.^22–26^ During operation, coordination of the *CO reaction intermediate increases the tendency for Cu migration, dissolution, and redeposition.^27–29^ The dynamicity of the Cu surface might initially yield a more active, roughened catalyst.^19,31^ However, continued restructuring inevitably leads to the loss of the desired catalyst layer roughness and activity over time, eventually impacting operational stability.^5,11,32^

Strategies to mitigate copper instability have been proposed, and each strategy comes with its own promise and challenges.^33–43^ Coating (e.g., porous oxides, carbon, organic layers) or alloying can enhance the structural stability of copper catalysts; however, they may suppress the overall activity by passivating and/or electronically modifying copper active sites.^32–34,40,42–47^ Pulsed operation has been shown to prolong the ethylene selectivity of Cu cathodes during CO_2_RR, yet the interplay with restructuring and the feasibility in large scale electrolyzers are open questions.^48–51^ Overall, space exists in the current state of the art for additional strategies that extend the operational stability of desired products without compromising the overall copper activity.

A few studies have highlighted the potential of strategies based on molecular surface chemistry to modulate catalyst reactivity in CO_2_RR.^31,52–56^ For example, the unique adsorption–desorption kinetics of phosphonate ligands were demonstrated to regulate and to enhance the CO_2_ to CO conversion on silver catalysts.^56,57^ Metal–ligand bond strength was identified to crucially determine the persistence of organic ligands on copper surfaces during CO_2_RR, hinting at their impact on Cu reconstruction.^55^ Yet, the direct link between molecular surface chemistry strategies and operational stability remains underexplored.

Here, we propose a molecular surface chemistry strategy that extends the operational stability of Cu electrocatalysts for ethylene production through a ligand-modulated release of copper surface sites. We introduce a surface chemistry approach that functionalizes the copper surface with either phosphonate or phosphine ligands. We discover that phosphines stabilize ethylene production compared to phosphonate and bare copper surfaces without compromising the selectivity and activity. Operando and ex-situ characterization link the desorption kinetics of the surface ligands to the activation of catalytic sites. Molecular dynamics simulations provide a more atomistic picture regarding such activation by clarifying the role of the ligands. Overall, the proposed molecular strategy has the potential to redefine the role of surface ligands in electrocatalysis, opening avenues for new catalyst design strategies toward long-term operation.

## Results and Discussion

### Surface Chemistry Development

We start by using well-defined colloidal Cu catalysts functionalized with one monolayer of ligands to establish the fundamental chemistry platform behind the proposed molecular strategy, before demonstrating its applicability to commercial Cu catalysts.

We synthesize Cu nanocrystals (NCs) with equal spherical morphology and size, yet two different surface functionalizations (Figure 1a), one with tetradecylphosphonic acid (TDPA) and one with diisobutylphosphine (PR_2_H), by manipulating synthesis chemistry (See Supporting Information S1 for details). We obtain the Cu-TDPA catalysts via a common route across the literature.^19,21,22,58,59^ Then, we introduce a novel synthesis route to obtain Cu-PR_2_H with the same size and morphology, which is crucial for a fair comparison of their CO_2_RR behavior. This novel synthesis route expands on a recently proposed CuBr-phosphine chemistry.^60^ The strength of this proposed approach lies in the ease with which the desired NC morphology and size can be synthesized (see Supporting Information S1 for details).

**Figure 1:**
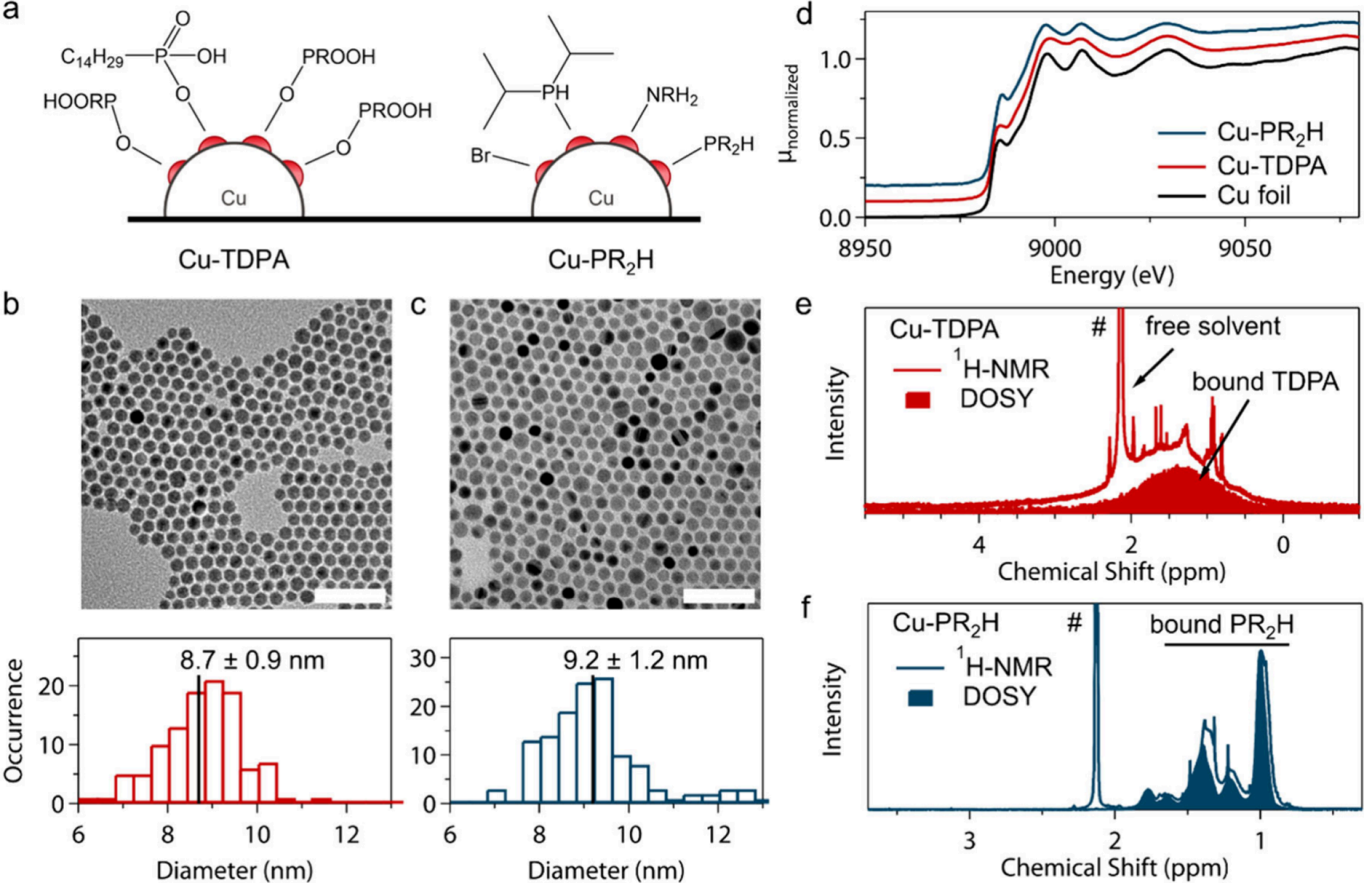
**Surface chemistry of Cu nanocatalysts obtained from customized synthesis routes.** (a) Ligand chemistry of Cu-TDPA and Cu-PR_2_H resulting from the multimodal characterization reported in this figure. Red semicircles represent Cu surface atoms. (b,c) TEM images and corresponding size histograms of Cu-TDPA (b) and Cu-PR_2_H (c). Scale bar = 50 nm. (d) XAS spectra of the Cu K-edge near-edge region. (e,f) Solution ^1^H NMR of purified colloidal dispersions of Cu-TDPA (e) and Cu-PR_2_H (f) NCs. The superimposed DOSY filter at 40% isolates slowly diffusing resonances of NC bound ligands. # indicates toluene solvent signals.

Transmission electron microscopy (TEM) images show that Cu NCs with the same spherical morphology and comparable average size of around 9 nm are obtained with TDPA and PR_2_H, respectively (Figure 1b,c). X-ray absorption spectroscopy (XAS) in the near edge region indicates that the as-synthesized Cu NCs are mostly metallic (>80%) based on comparison with Cu foil (Figure 1d, Figure S3). Proton nuclear magnetic resonance (^1^H NMR) spectra along with the superposed diffusion ordered spectroscopy (DOSY)-filtered spectra (Figure 1e,f), the 2D-DOSY maps (Figures S4, S5), and X-ray photoelectron spectroscopy (XPS) (Figures S6) provide a complete picture of the surface chemistry for Cu-TDPA and Cu-PR_2_H. The ^1^H NMR spectra feature broadened resonances (Figure 1e,f), which are consistent with surface bound ligands in nanocrystal dispersions.^61^ The single large and broadened peak in the superposed DOSY-filtered spectrum for Cu-TDPA corresponds to the protons in the saturated aliphatic backbone of the phosphonate ligand (Figure 1e, Figure S4). Peaks attributable to the protons of the secondary phosphine emerge as the major contribution to the signal for Cu-PR_2_H (Figure 1f, Figure S7 **).** Surface analysis revealed the presence of oleylamine (14%) and bromine (28%) on Cu-PR_2_H, although as a minor fraction compared to diisobutylphosphine (58%) (Figure S6, S7). Quantitative NMR in combination with mass spectrometry indicated a ligand density of 4.3 molecules/nm^2^ for Cu-PR_2_H, and XPS confirmed a comparable ligand density on Cu-TDPA as derived from an equivalent P:Cu ratio (Figure S6).

### Impact of Surface Chemistry on CO_2_RR Performance

Having obtained Cu catalysts of comparable morphology, size, and ligand density, we tested the CO_2_RR performance of the Cu-TDPA and Cu-PR_2_H along with those of commercial Cu with added diisobutylphosphine to identify the impact of the proposed surface chemistry on the CO_2_RR performance (Figure 2). We chose to add only the diisobutylphosphine to the commercial Cu sample for simplicity and given that this ligand represented the major surface component in Cu-PR_2_H.

**Figure 2:**
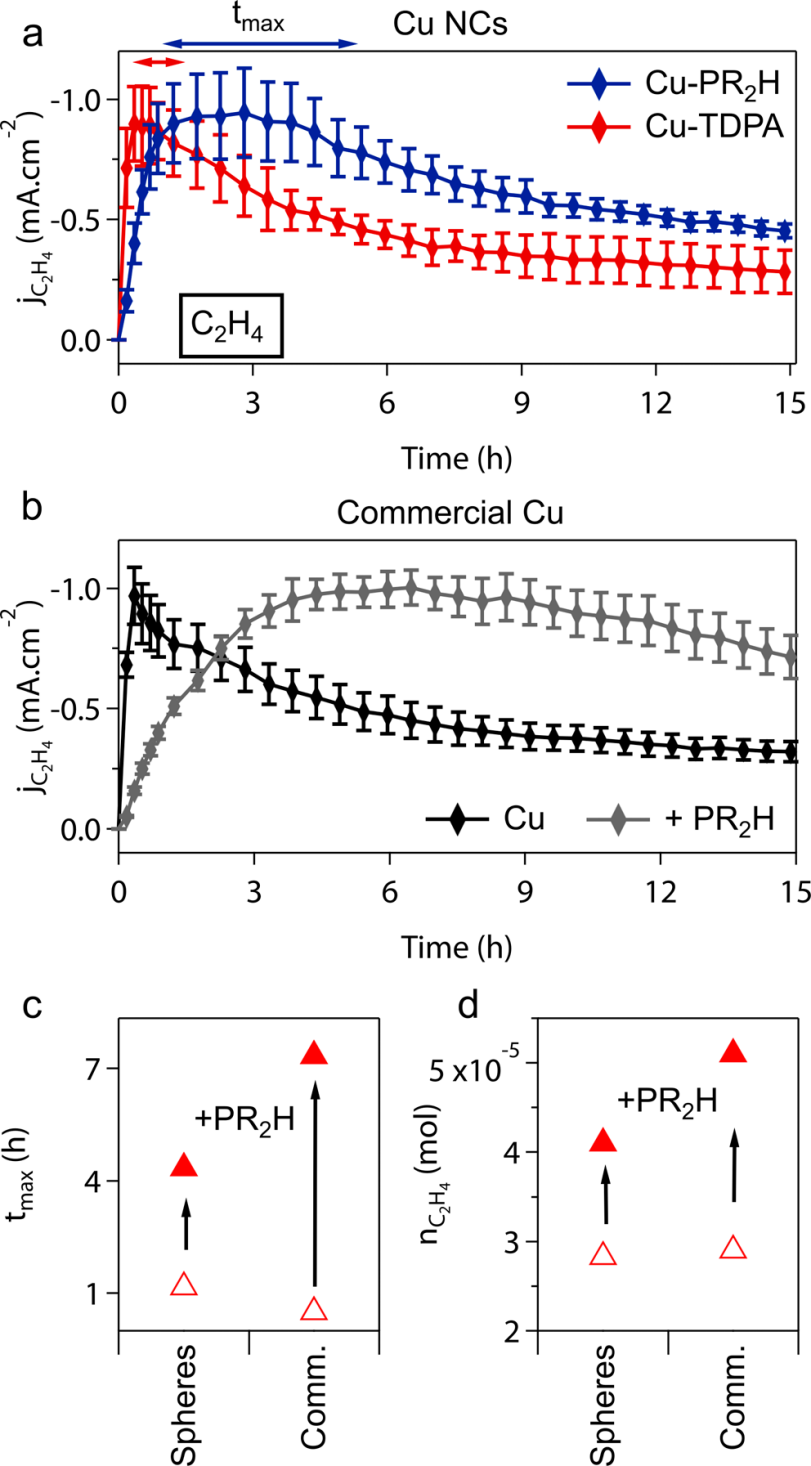
**CO_2_RR performance of Cu nanocatalysts and commercial Cu with different surface chemistries.** (a) Ethylene partial current densities (*j*_C2H4_) for Cu-TDPA and Cu-PR_2_H NCs as a function of operation time. (b) Ethylene partial current density (*j*_C2H4_) for commercial Cu prepared with Nafion and with the addition of PR_2_H. Data points are the averaged current density per 30 min over 3 separate runs with the same catalyst. Error bars are the standard deviation. (c) Time at which 90% of the maximum ethylene current density is produced, *t*_max_. (d) Total amount of ethylene produced during 15 h of continuous CO_2_RR with Cu NCs and commercial Cu upon modification with secondary phosphine ligands. These data were acquired at −1.2 V_RHE_ in 0.1 M KHCO_3_ by drop-casting the Cu catalysts on flat glassy carbon electrodes. This potential was selected to maximize ethylene current density based on previous studies with Cu NCs.^9^

We utilized a H-type cell, which facilitates identifying and isolating catalyst failure with negligible effects of catalyst loading, salt formation and membrane failure present in high current density devices.^4^

Cu-TDPA and Cu-PR_2_H NCs have similar average CO_2_RR performance in terms of current density and product selectivity after the first hour of operation (Figure S8). Instead, intriguing differences emerge when monitoring the CO_2_RR performance over a longer time (Figures 2a, S9, S10). Herein, we focus on ethylene production rate (*j*_C2H4_), being the main product of interest (Figure 2a), while we discuss the production rate for hydrogen and methane (*j*_H2_ and *j*_CH4_, respectively) in the Supporting Information (Figures S10, S11)

First, we note that *j*_C2H4_ initially increases for both Cu-TDPA and Cu-PR_2_H and reaches a maximum before starting to decrease. Cu-PR_2_H reaches this maximum *j*_C2H4_ later than Cu-TDPA (3 h vs 20 min) and sustains it for a longer time compared to Cu-TDPA. Indeed, the time window for which 90% of the maximum *j*_C2H4_ is maintained (*t*_max_) for Cu-PR_2_H is more than 4 times longer compared to that measured for Cu-TDPA (Figure 2a).

Even more dramatic results are obtained with commercial Cu catalysts upon addition of PR_2_H to the catalyst ink (Figure 2b, Figure S11). The commercial Cu catalyst achieves maximum *j*_C2H4_ within 20 min and rapid deactivation follows (Figure 2b), which resembles Cu-TDPA (Figure S12). Impressively, the addition of 0.5 equiv of PR_2_H per Cu surface atoms to the catalyst ink (i.e., ligand density of 5 molecules/nm^2^, similar to the synthesized Cu NCs) slows down the activation (maximum *j*_C2H4_ achieved in 4 h) with 10 times prolonged maximum *j*_C2H4_. Excess ligands (2 equiv) provide even greater current density stability, although at a penalty in overall activity (Figure S13). Overall, the phosphine functionalization results in Cu catalysts with a prolonged selectivity for ethylene (Figure 2c), which leads to a greater amount of ethylene produced during the 15 h experiment with the same mass of Cu (Figure 2d).

We further confirmed that diisobutylphosphine coordinates to the surface of commercial Cu by using Fourier transform infrared spectroscopy (FTIR) (Figure S14). In addition, we demonstrated that injections of the ligand into the electrolyte do not interfere with the catalytic performance (Figure S15).

Altogether, the data above confirm that the adsorbed secondary phosphine plays a central role in extending the operational stability of ethylene production, and the replicability on commercial Cu hints at the dominant role of the phosphine in the mixed ligand shell of the Cu-PR_2_H NCs.

### Impact of Surface Chemistries on Compositional and Morphological Changes of the Catalysts

Having assessed the impact of the TDPA and PR_2_H on the CO_2_RR performance and observed the prolonged ethylene production with PR_2_H, we employed a suite of in situ spectroscopic tools and ex-situ microscopy to correlate the temporal catalytic differences to the evolving catalyst surface chemistry, copper oxidation state, and morphology.

We analyzed the evolution of the surface ligands during CO_2_RR using attenuated total reflection Fourier transform infrared spectroscopy (ATR-FTIR) with a customized setup (Figure 3a, Figures S16, S17 and Supporting Information S4 for details). The FTIR spectra of both Cu-TDPA and Cu-PR_2_H show characteristic resonances of the ligand moieties, along with resonances from water and carbonate upon immersion in the electrolyte. The C–H stretch vibrations between 2800 and 3100 cm^–1^ are ideal to track the fate of the ligands (i.e., adsorption/desorption) because they only interfere with the water band, which can be easily subtracted (Figure S18, S19). Thus, we used the relative integrated absorbance of the C–H vibrational resonances as an estimate of the surface coverage during electrochemistry.

**Figure 3:**
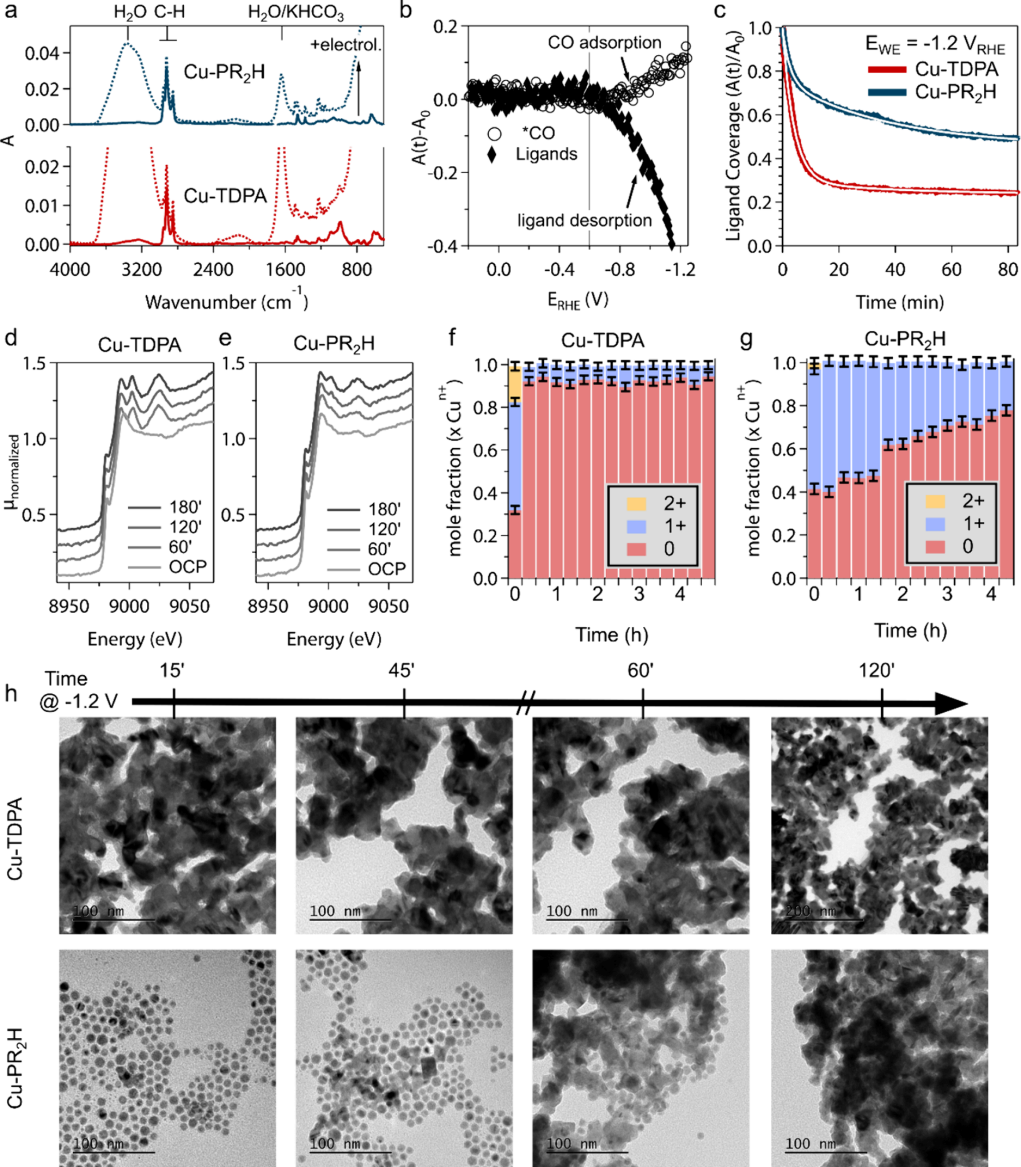
**Operando and ex-situ characterization linking ligand chemistry, copper oxidation state, and morphological changes.** (a) ATR-FTIR spectra of dropcasted Cu NCs on graphene as a dry film (solid) and immersed in KHCO_3_ electrolyte (dashed line). (b) Integrated differential absorbance during the start-up (i.e., linear sweep voltammetry) of Cu-TDPA. The onset of CO adsorption and CO_2_RR coincides with ligand desorption. (c) Evolution of the surface ligand coverage as estimated by the relative integrated absorbance of the C–H stretching vibrations *A*(*t*)/*A*_0_. (d, e) Operando XANES spectra at 60 min intervals during CO_2_ reduction at −1.2 V vs RHE for Cu-TDPA (d) and Cu-PR_2_H (e). (f, g) Linear combination analysis results demonstrate the Cu speciation as a mole fraction *x* during 4 h of CO_2_ reduction for Cu-TDPA (f) and Cu-PR_2_H (g). (h) Representative TEM images taken after 20, 40, 60, and 120 min of CO_2_RR for Cu-TDPA and Cu-PR_2_H catalysts on glassy carbon.

We found that an interplay between ligand desorption and *CO adsorption exists during start-up (Figure 3b, Figure S20). Specifically, the *CO peak at 2030 cm^–1^ starts to appear and simultaneously ligands desorb as evidenced by reduced absorbance of the C–H vibrations (Figure 3b**)**. These data indicate that surface activation for the CO_2_RR follows initial ligand desorption.

Next, we addressed the kinetics of ligand desorption for Cu-TDPA and Cu-PR_2_H samples during the first 1.5 h of operation (Figure 3c). Cu-TDPA shows faster ligand desorption compared to Cu-PR_2_H and, additionally, loses more than 75% of ligands, compared to 50% on Cu-PR_2_H within 1.5 h (Figure 3c). The desorption kinetics on both model systems can be fit with two-site adsorption kinetics, where the dominant component in Cu-PR_2_H is roughly a factor 10 slower than in Cu-TDPA (Figure 3c, Figure S21).^62^ The two site-adsorption kinetics indicate heterogeneity of adsorption sites on the NC surface (e.g surface facets, surface defects).^62^ We could speculatively attribute the different adsorption sites to the (100) and (111) facets on the spherical NCs; however, a dedicated experimental-theoretical study would be necessary. Notably, the diisobutylphosphine and the oleylamine desorb at the same time on Cu-PR_2_H (Figure S22). These data, along with the results on the commercial Cu sample, strengthen the idea that the contribution from oleylamine, if any, is minimal. This interpretation is further supported by previously published results.^63–66^ Previous studies on cubic Cu NCs with oleylamine and bromine used in the synthesis report no delayed activation for ethylene production.^63,64^ Additionally, the impact of halides on Cu reactivity has been reported, however only when halides were present in the electrolyte at many orders of magnitude greater concentration than those adsorbed on the surface of the Cu-PR_2_H NCs.^65,66^

We followed the eventual impact of ligand coordination on the copper oxidation state during CO_2_RR with operando X-ray absorption near edge structure (XANES) spectra (Figure 3d-g, Figure S23). The Cu-TDPA and Cu-PR_2_H start in a similarly oxidized state at open circuit potential, which results from drop-casting in air and immersion in the electrolyte, in line with several previous studies.^21,22,33,34^ Cu-TDPA reduces to metallic copper as soon as the cathodic bias is applied and remains metallic over time with some residual oxide, which cannot be distinguished from the experimental and fitting error. On the contrary, the reduction kinetics of Cu-PR_2_H are much slower with 20% of oxidized Cu persisting even after 4 h of operation.

Finally, we monitored the morphological changes of the catalysts via ex-situ TEM. (Figure 3h, Figure S24). Cu-TDPA shows complete reconstruction already after 15 min of CO_2_RR, which is consistent with many reports in the literature.^22,33,34^ On the contrary, the reconstruction kinetics of Cu-PR_2_H are much slower. Indeed, isolated spherical copper NCs are still distinguishable over the first hour of operation and the Cu-PR_2_H resembles the restructured Cu-TDPA catalyst only after 2 h. Morphologically more stable Cu catalysts are characterized by persistent cationic copper sites.^33,34^ Thus, these data correlate well with the higher oxide fraction from operando XANES.

### Simulating the Effect of Ligands on Cu Surface Reactivity

Prior studies link the presence of cationic-rich copper surfaces and of grain boundary-rich domains to ethylene-selective active sites.^19,21,58,67–70^ In our experiments, ethylene production follows that of ligand desorption. Reduction of cationic copper to metallic copper and formation of grain boundaries accompany ligand desorption, which hints at a stronger link between the formation of ethylene-selective active sites and the formation of grain boundaries, rather than the presence of cationic copper.

In addition, we explored the possibility that the phosphines contribute to the Cu reactivity more directly via their surface binding using simulations (Figure 4).

**Figure 4:**
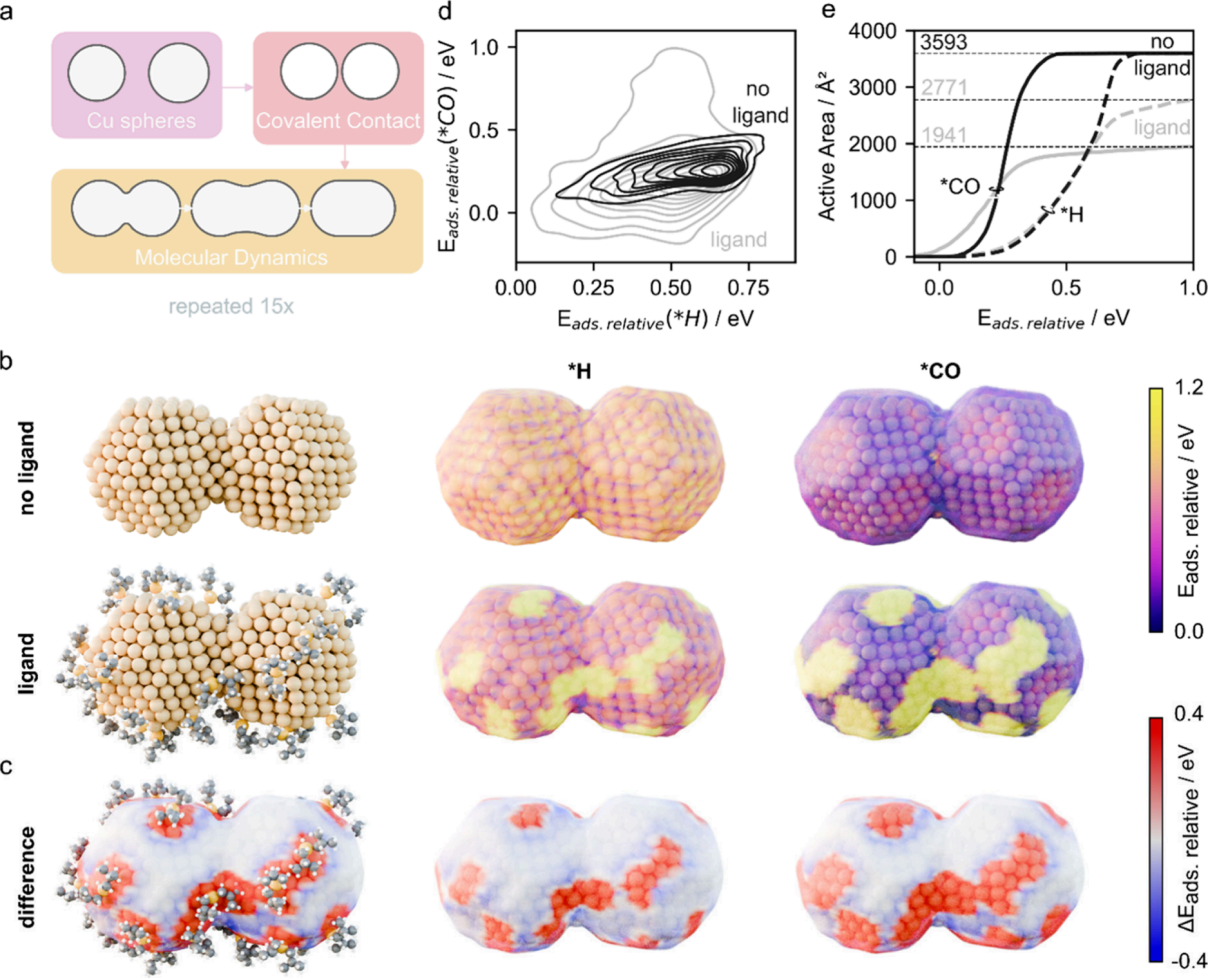
**Atomistic simulation of ligand-passivation on grain boundary forming Cu NCs.** a) Workflow for simulating grain boundary between Cu NCs. Cu spheres are generated, and their relative orientation is randomized. Their position is set to first covalent bond distance, relaxed, and evolved with molecular dynamics. b) Atomistic structure of the roughened interface (MD snapshot) between two Cu spheres simulating grain boundaries along with superposed color maps reflecting intermediate adsorption energies, wherein lower *E* values reflect more favorable binding sites. c) Local effect of the ligands on the adsorption energy of CO and H with and without ligands along with superposed color maps reflecting the relative intermediate adsorption energies, wherein negative Δ*E* reflects more favorable binding sites. d) Contour plots indicating the distribution of adsorption energy of *CO versus *H with and without ligands.^75^ e) Total available active area for *CO and *H adsorption with and without ligand coverage. Adsorption sites for *CO are more significantly suppressed by ligands than by *H sites.

Atomistic models of the size and complexity required to capture the behavior of real systems have only recently become computationally tractable using machine learning interatomic potentials, (MLIP)^71^ allowing us to generate a representative, nanostructured model catalyst wherein two spherical Cu NCs come into contact, simulating grain boundary formation (Figure 4a, see Supporting Information S6 for details).

Following the simulated grain boundary formation, we kept the position of the Cu atoms constant as we evaluated their affinity for intermediate adsorption in the presence and absence of ligands (Figure 4b,c). We selected the ligand coverage/density to correspond to roughly 60% of maximum coverage to reflect the initial stages of catalyst activation and compare it to a ligand free surface, representative of prolonged operation.

We chose *H and *CO as intermediates, as they are crucial to determine pathways in CO_2_RR and in the competing HER.^72–74^ We compared their relative adsorption energies (*E*_ads relative_) on a 0 to 1 eV scale (Figure 4b, d), where the most favorable adsorption site is set to 0, and on a relative scale, defined as the difference in adsorption energy between ligand and ligand-free particles (Δ*E*_ads relative_), wherein the most favorable adsorption sites correspond to negative Δ*E* (Figure 4c). In addition, we compared the total available active areas for *CO and *H adsorption with and without ligand coverage (Figure 4e). Steric repulsion of the side chain and increased covalent affinity of neighboring Cu sites emerged as the main local effects of the ligands on intermediate adsorption (see Methods for details).

The energy landscape of the ligand-free Cu surface is overall uniform with a slightly enhanced affinity (dark purple region) for *CO at the grain boundary between two particles (Figure 4b, Figure S25). The ligands introduce site heterogeneity, wherein the ligand-free sites possess enhanced relative adsorption energy for both *H and *CO, and the regions at the ligand site show reduced affinity (Figure 4b). Higher affinity of ligand-free sites is further emphasized by the broadened distribution of adsorption energies for *CO and *H in the presence of ligands (Figure 4d). However, the area of the region with a higher affinity (lower *E*_ads_) for *H is larger than the region with an enhanced affinity for *CO, which becomes more evident when plotting the relative difference in adsorption energies with and without ligands (Figure 4c) and in comparison to roughened Cu sites. The reduced active area can be seen graphically from the cumulative area as plotted versus the adsorption energy (Figure 4e). These data indicate that the phosphine ligands reduce the surface active area for *CO to a larger extent than for *H, yet they increase the affinity of the exposed sites for *CO. Since C–C coupling requires adjacent *CO intermediates, the reduced *CO site density likely disfavors ethylene formation. The simulations are consistent with experimental observations showing methane-dominated selectivity during the early stages of catalyst activation (Supplementary Figures S11) when a more substantial fraction of the phosphines remain on the surface. Phosphine ligands induce pronounced site heterogeneity, passivating large surface areas while enhancing *CO and *H binding at exposed Cu sites, yet disproportionately reducing the density of *CO adsorption sites required for C–C coupling. Consequently, ethylene-selective active sites emerge only after ligand removal, highlighting the dual kinetic and surface-binding roles of phosphines in governing catalytic selectivity.

### Ligand-Modulated Catalyst Activation

The results presented above clearly link the surface ligand chemistry with the compositional and structural evolution of the Cu catalysts as well as ethylene active site evolution and ethylene production (Figure 5).

**Figure 5:**
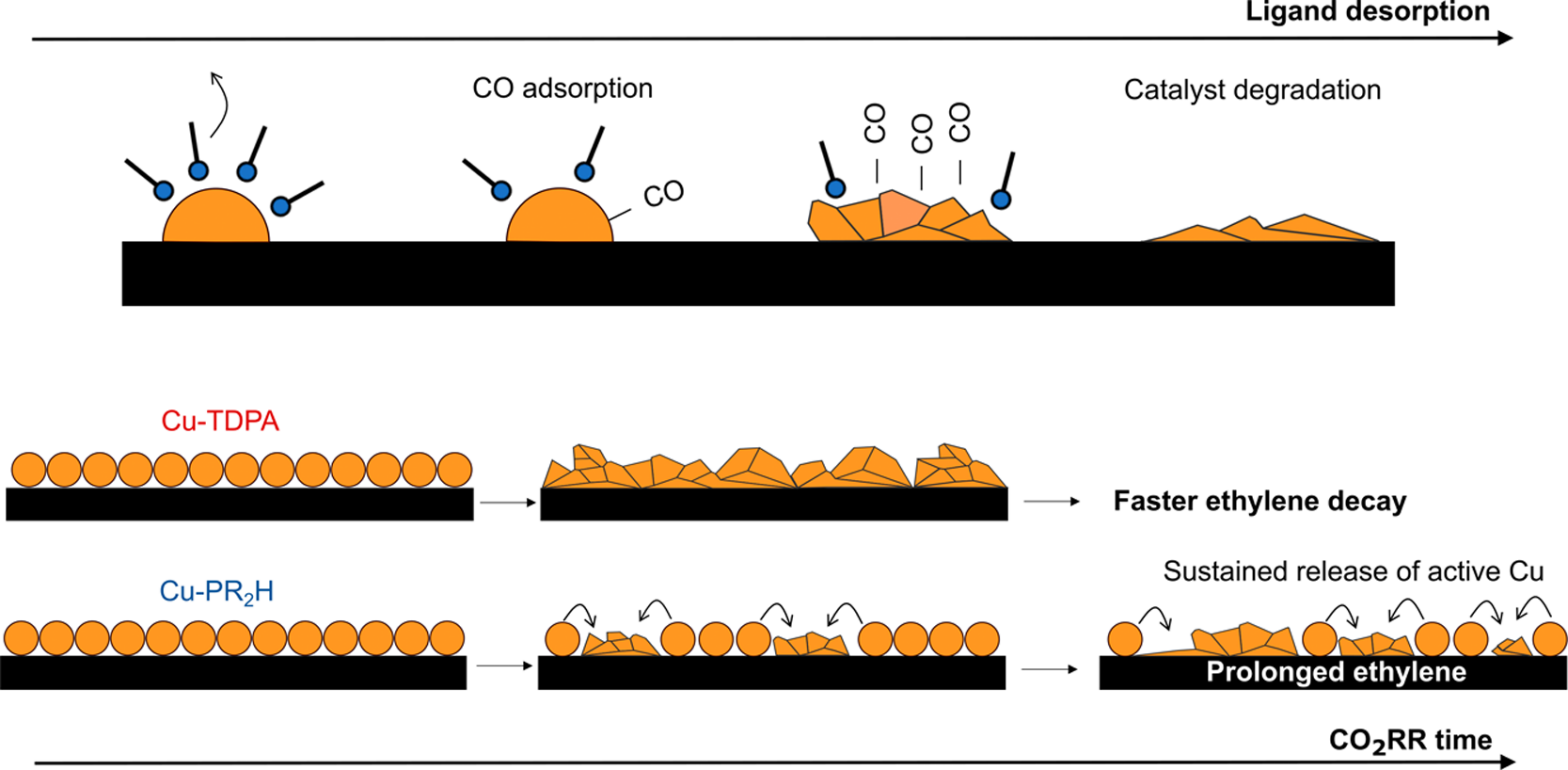
**Ligand-modulated copper catalyst activation and ethylene selectivity during the CO**_**2**_ **RR.** Ligand desorption occurring during the cathodic potential ramp enables CO adsorption, which we connect to CO_2_RR activation. Ligand desorption releases ethylene-active sites via modulating Cu reconstruction and reduction during operation. The slower desorption kinetics of PR_2_H compared to TDPA enable a prolonged ethylene selectivity via a sustained release of the catalytically active sites.

First, the CO adsorption onset overlaps with the ligand desorption onset during the initial cathodic ramp, hinting at an initial surface activation being needed. While surface ligands do not suppress surface reactivity altogether, ligands alone do not promote ethylene selectivity. Instead, the progressive desorption of ligands modulates the release of ethylene active sites forming from copper reconstruction and grain boundaries formation. The released active surface eventually evolves into a surface inactive for ethylene after complete ligand desorption.

While both Cu-TDPA and Cu-PR_2_H catalysts ultimately achieve similar ethylene activity, their desorption kinetics differ markedly. Binding energy, electrostatic repulsion, and steric hindrance are all contributing factors to this different behavior.^52,54,57,76^ Phosphonate ligands (TDPA) desorb more rapidly under a cathodic bias. A stronger contribution of the electrostatic repulsion between the X-type ligand’s anionic headgroups and the negatively polarized electrode might explain this behavior.^73,74^ The secondary phosphine (PR_2_H) is a softer, neutral L-type ligand, which is expected to more strongly bind to soft Cu^+^ atoms, and desorbs more gradually in the absence of electrostatic repulsion.^77,78^ The emerged differences between X- and L-type ligands, as well as binding energy differences implied from Pearson’s hardness, inspire future studies to define a pathway toward further optimization of ligand desorption kinetics in larger ligand libraries.

The slow desorption of PR_2_H delays the time at which the maximum level of ethylene production is reached. By slowing down activation, the window during which ethylene-selective sites are formed and maintained is extended in Cu-PR_2_H. Akin to sustained release formulations, we speculate that active sites are continuously formed and degraded sites are replaced.

### Stability and Electrolyzer Configuration

The 10-fold improvement factor in operational stability obtained via manipulating the ligand desorption kinetics, herein using diisobutylphosphine, is comparable to the relative improvement obtained by other microenvironment manipulation (i.e., introducing hydrophobic layers) or pulse methods to reach a hundred hours of operation at high current densities.^48,79,80^ Toward this practical milestone, our model chemistry must first be demonstrated in a membrane electrode assembly (MEA).^81^ The specific ligand chemistry might vary because of the greater current density and potential difference as well as the existence of a three-phase boundary in MEA devices.^82^ However, the strategy of using ligands to achieve sustained release of active sites should be universal, and we anticipate that the chosen ligand will have to match the cell-specific catalyst degradation kinetics. Also here, operando studies in the corresponding cell design will be essential to develop.

## Conclusions

In this work, we propose a molecular surface chemistry strategy to modulate Cu catalyst activation and ethylene operational stability in CO_2_RR by tuning surface ligand chemistry. Specifically, we discovered that secondary phosphines desorb more slowly from Cu during electrocatalysis, delaying Cu reduction, restructuring, and the onset of maximum ethylene production. This kinetic control extends the period of maximum ethylene output by up to ten times compared to bare or phosphonate-covered Cu catalysts.

We interpreted the prolonged stability of ethylene production of the phosphine-functionalized catalyst as arising from slower activation, modulated by ligand desorption, which acts as a sustained-release mechanism for generating and maintaining active sites for C–C coupling. This approach does not alter the product selectivity or activity of the copper catalyst but significantly enhances operational longevity.

This insight reframes the scope of surface ligands in electrocatalysis. Rather than directly modulating the nature of the catalytic site, ligands here function as kinetic regulators that control the release and lifetime of active surface sites. This ligand-governed restructuring rate introduces a new design parameter for catalysts aimed at long-term operation. The sustained release concept could be applied to other electrocatalysts that undergo operando activation, offering a route to align the catalyst lifetime with that of other device components, minimizing downtime, reducing catalyst loss, and increasing system efficiency.

## Supplementary Material



## Data Availability

All data are available in the main text or the Supporting Information. Experimental data are openly available in Zenodo at: 10.5281/zenodo.18798600.

## Notes

Supplementary methods, additional details concerning Cu NC synthesis with phosphine ligands, XAS fitting, XPS and NMR characterization, supplementary electrochemistry data, details concerning the ATR-FTIR experiments, post-mortem TEM analysis, and discussion concerning the simulation platform and results (PDF)
