# Supplementary material for: Ligand-Modulated Release of Copper Active Sites Extends Ethylene Production in CO2 Electroreduction
Source: J Am Chem Soc. 2026 Mar 19;148(12):13118–27. doi: 10.1021/jacs.5c22701 (PMC13047531; doi:10.1021/jacs.5c22701)
Supplement: Supplementary file 1 [file ja5c22701_si_001.pdf]

Supporting Information

Ligand-Modulated Release of Copper Active Sites Extends Ethylene Production in CO_2 Electroreduction

Jari Leemans[1], Edvin Fako[2], Ludovic Zaza[1], Moritz Tritschler[1], Junwu Chen[2], Philippe Schwaller[2], Raffaella Buonsanti[1,*]

1. Laboratory of Nanochemistry for Energy, Institute of Chemical Sciences and Engineering, École Polytechnique Fédérale de Lausanne, 1950 Sion, Switzerland

2. Laboratory of Artificial Chemical Intelligence, Institute of Chemical Sciences and Engineering, École Polytechnique Fédérale de Lausanne, 1015 Lausanne, Switzerland

Raffaella.buonsanti@epfl.ch

Experimental Section **3**
S1 Synthesis advancement towards spherical Cu nanocrystals with phosphines **6**
S1.1 Altering reaction kinetics with secondary phosphine ligands 6
S1.2 The synthesis of spherical sub-10 nm nanocrystals with di-isobutylphosphine 6
S2 Characterizing the nanocrystals **9**
S2.1 Linear combination fitting of XANES spectra 9
S2.2 Diffusion ordered nuclear magnetic resonance spectroscopy (NMR) 10
S2.3 Assigning proton resonances in Cu-PR_2H 10
S2.4 Quantifying the relative composition of ligands on Cu nanocrystals 12
S3 Additional electrochemistry data **14**
S3.1 Selectivity of nanocrystal catalysts after 1 hour of CO2RR 14
S3.2 Faradaic efficiency of gas product generation with Cu nanocrystals 15
S3.3 Selectivity of commercial Cu electrodes modified with Nafion and additives 17
S4 ATR-FTIR **21**
S4.1 Benchmarking graphene on Si ATR crystal electrodes 21
S4.2 Confirming ligand identification from ^{1}H-NMR results with FTIR 22
S4.3 Quantifying ligand desorption 23
S4.4 CO adsorption, identifying the onset of CO_2RR 24
S4.5 Fitting ligand desorption with two-site kinetics 25
S4.6 The desorption lineshape on Cu-PR_2H 26
S5 Operando and post-mortem characterization **27**
S5.1 Operando x-ray absorption spectroscopy and linear combination analysis 27
S5.2 Additional post-chronoamperometry electron microscopy 28
S6 Simulations to probe ligand effects in CO2RR intermediate adsorption **29**
S6.1 Supplementary Discussion 29
S6.2 Computational Details 30
References **35**

Experimental Section

Chemicals:

Copper(I) Acetate (Cu(I)OAc, 98%), CuBr (99.999% trace metals basis), Cu (nanopowder, 25 nm), tri-n-octylamine (technical grade, 98%), oleylamine (technical grade, 70%), tetradecylphosphonic acid (97%), toluene (anhydrous, 99.8%), ethanol (dried, max 0.01% H_2O) and toluene-d_8 were purchased from Sigma-Aldrich Chemie GmbH. Di-isobutlyphosphine (97%) was purchased from ABCR Swiss *AG*. K_2CO_3 (99+%) was purchased from Thermo-scientific. Gaseous CO_2 (50, 99.999%) was purchased from Carbagas *AG.*

General synthetic considerations

All syntheses and manipulations of Cu nanocrystals (NCs) were performed under a dry N_2 atmosphere, using Schlenk-line techniques or a glovebox. Anhydrous organic solvents were used for the manipulation, analysis, and storage of the NCs. All glassware was oven-dried prior to use. Concentrated nitric acid was used to remove any metallic residues from the reaction flask after each reaction, and the flask was then washed thoroughly with ultrapure water prior to oven drying. A J-KEM Scientific model 310 temperature controller was used with a heating mantle for reaction temperature control.

Synthesis of spherical Cu nanocrystals with tetradecylphosphonic acid/trioctylamine (Cu-TDPA)

20 mL of trioctylamine is degassed under vacuum for 1 hour at 120°C connected to a Schlenk line in a three-neck flask equipped with stirring bar and heating mantle. After degassing, the flask is filled with N_2 and cooled to 50°C. The septum is opened and 272 mg (1 mmol) tetradecylphosphonic acid and 242 mg (2 mmol) Cu(I)OAc are added to the three-neck flask. The reaction mixture is heated to 180°C under N_2. After 30 minutes at 180°C the temperature is increased to 270°C. After 30 minutes at 270°C the reaction mixture is cooled by removing the heating mantle. The deep red dispersion is transferred air-free to an inert atmosphere glovebox. The Cu nanocrystals are isolated by three consecutive cycles of precipitation/redispersion with toluene and ethanol. The final pellet is dispersed in 5 mL of toluene to yield a concentrated stock solution.

Synthesis of spherical Cu nanocrystals with oleylamine/di-isobutylphosphine (Cu-PR_2H)

In a glovebox, copper(I) bromide powder (65 mg, 450 μmol) and di-isobutlyphosphine (80 μL, 450 μmol) were added to a 3-neck flask. Afterward, a stirring magnet and 14 mL of predegassed oleylamine were added. The flask was sealed and quickly connected to a Schlenk line under N_2. The reaction mixture was then degassed for 5 min at 60°C before being heated for 20 min under N_2 at 200°C with a heating ramp of ~20°C/min. The solution, originally reddish, turned brown at 200°C. The 20 min reaction was started once the temperature reached 200°C. At the end of the reaction, the reaction mixture was cooled down to approximately 80°C before being transferred to the glovebox. The reaction mixture

was washed and centrifuged 2 times at 13,000 rpm for 10 min, the first time with 15 mL of hexane and the second time with 5 mL of hexane and 5 mL of ethanol. At the end, the final product was collected in 1 mL of toluene for further analysis.

Electrochemical experiments

Electrochemistry was performed in a custom 4 mL internal volume polycarbonate H-type with catholyte and anolyte compartments separated by a Selemion AMV anion-exchange membrane. The cathode and anode are oriented in parallel fashion. 0.1 M $KHCO_3$ solution was used as the electrolyte. A Biologic SP-300 potentiostat was connected to the glassy carbon working electrode, the platinum foil counter electrode and the Ag/AgCl reference electrode. CO_2 was bubbled through both anolyte and catholyte compartments with a frit-fitted gas inlet. To study the synthesized nanomaterials, thin films were prepared by dropcasting 15 micrograms of Cu, onto the 1.33 cm^2 active area of the glassy carbon working electrode.

In contrast to the colloidal nanocrystals, commercial Cu catalyst electrodes were prepared by dropcasting a dispersion containing 1 mg/mL Cu and 0.05 mg/mL Nafion in dry isopropanol. In the case where di-isobutylphosphine was added, the dispersion was made air-free and sonicated in a purged vial to prevent ligand oxidation prior to electrode preparation. The final Cu loading on the electrode was 40 micrograms per cm^2.

Samples were tested for the CO_2 reduction reaction (CO_2RR) with the following protocol. First, electrochemical impedance spectroscopy was performed to estimate the uncompensated resistance in the cell. Dynamic iR-compensation at 85% was consequently applied to all experiments. Then the sample was pre-conditioned with a single linear sweep voltammetry scan from open circuit potential to -2 V vs Ag/AgCl. After a wait period of 2 minutes, a series of cyclic voltammetry experiments were performed with a scan window of 100 mV around -0.4 V vs Ag/AgCl and increasing scan rates to calculate the double layer capacitance, from which the electrochemical surface area can be estimated. Then, chronoamperometry at the desired potential was performed with a constant CO_2 flow rate of 5 sccm. Following the chronoamperometry, another double layer experiment was performed to estimate the double layer capacitance after electrolysis.

Gas products were separated and analyzed with the use of a gas chromatograph (GC, SRI Instruments) equipped with a HayeSep D porous polymer column, thermal conductivity and flame ionization detectors. The exhaust of the catholyte compartment is directly connected to the inlet of the GC. faradaic efficiency of the gaseous products is calculated by establishing a calibration curve for the GC detectors with the use of standard gas mixtures (Carbagas).

Liquid products are isolated by sampling the electrolyte post-electrolysis. Liquid products are separated and quantified with the use of high-performance liquid chromatography and a refractive index detector.

A Aminex HPX-87H (BioRad) column and a 5 mM H_2SO_4 eluent were used to separate the carboxylic acid and alcohol products. A calibration curve to quantify ethanol, n-propanol, ethylene glycol, formic acid and acetic acid was established by mixing a series of calibration standards in a 0.1 M $KHCO_3$ electrolyte.

Electron Microscopy

Sample preparation of the purified nanoparticles consisted of drop-casting 10 microliters of the respective dispersions onto a Cu grid. Post-electrolysis electron microscopy sample preparation was performed by rinsing the electrode with water, consequently drop-casting 10 microliters of toluene onto the dry electrode surface and scraping the catalyst residue onto the TEM grid through the toluene. Bright-field electron microscopy was performed on a ThermoFisher Scientific Tecnai G2 Spirit Twin at 120 kV equipped with a Lens-coupled 4-megapixel Gatan Orius SC200D camera.

X-ray photoelectron spectroscopy

XPS spectra were recorded using an Axis Supra (Kratos Analytical) instrument, using the monochromated Kα X-ray line of an Al anode. The pass energy was set to 20 eV with a step size of 0.1 eV. The samples were electrically insulated from the sample holder and charges were compensated. Spectra were calibrated to 284.8 eV at the C 1s orbital. Cu NCs samples were prepared by drop-casting nanocrystal films onto clean Si substrates.

Nuclear magnetic resonance spectroscopy

1D ^1H and ^{31}P-nuclear magnetic resonance spectra were recorded on a Bruker Avance IIIHD-400 spectrometer equipped with a $BBFO_2$ probe. Quantitative ^1H-spectra were recorded with 90 degree pulsing and 45 second waiting times between spectra to ensure complete relaxation, and ligand concentrations were obtained following the PULCON procedure. All spectra are recorded in deuterated toluene.

Inductively coupled plasma optical emission spectroscopy

Quantification of Cu concentration was performed by inductively coupled plasma optical emission spectroscopy on an Agilent ICP-OES 5110 instrument. Colloidal dispersions were sampled, the dispersing solvent was dried under N_2 flow and the remaining powder was consequently digested in concentrated nitric acid. After dilution, the samples as well as a series of calibration standards prepared from standard reference solutions of each element were injected into the instrument. Concentrations were determined from the ensuing calibration curve.

Supplementary Data

S1 Synthesis advancement towards spherical Cu nanocrystals with phosphines

S1.1 Altering reaction kinetics with secondary phosphine ligands

One versatile synthetic scheme to obtain different well-defined Cu nanocrystals (NCs) involves heating a copper(I) halide precursor with a phosphine ligand in oleylamine.[1–6] Cu(0) is formed from the disproportionation reaction of the in-situ generated molecular complex containing the copper(I) halide and phosphine ligand.[2,3]

In a previous study, we reported that the presence of P–H bonds in the phosphine ligand as a possible critical feature in the phosphines to accelerate the disproportionation reaction and widen the reaction temperature range that can be employed to generate Cu NCs.[2] In contrast to tertiary phosphines and tertiary phosphine oxides such as trioctylphosphine (TOP) and trioctylphosphine oxide (TOPO) typically used for Cu NC synthesis at > 250°C, secondary phosphines (i.e. PR_2H) allow to instantaneously form Cu(0) at 200°C while primary phosphines (i.e. PRH_2) allow to form Cu(0) at temperatures as low as 120°C.[2] We showed that Cu cubes mixed with various Cu twinned NCs were obtained after a 20-minute reaction at 200°C with PPh_2H , while spherical NCs were obtained with $P(^iBu)_2H$ at 200°C.[2]

S1.2 The synthesis of spherical sub-10 nm nanocrystals with di-isobutylphosphine

Our previous work identified di-isobutylphosphine as a promising ligand to obtain spherical ~10 nm Cu NCs,[2] which are comparable to those obtained through the TDPA route.

In this work, we synthesized 9.2 ± 1.2 nm Cu spheres with di-isobutylphosphine by using copper(I) bromide (CuBr) as the copper source and oleylamine (OLAM) as the solvent (**Figure 1b**). We used a 1:1 stoichiometry between CuBr and $P(^iBu)_2H$ and the Cu nanocrystals were obtained by heating the reaction solution during 20 minutes at 200°C.

When di-isobutylphosphine is mixed with CuBr in OLAM, a colored red-orange solution is obtained initially, which then turns dark around 200°C upon the formation of Cu NCs (**Figure S1**). The initial red color is indicative of the formation of a molecular complex between CuBr and $P(^iBu)_2H$, similarly to what is observed with PPh_2H. When PPh_2H is mixed to CuBr in a 2:1 stoichiometry in OLAM, a bright yellow solution is initially obtained with the formation of bimetallic$\{CuBr(PPh_2H)_2\}_2$ molecular complex.[2]

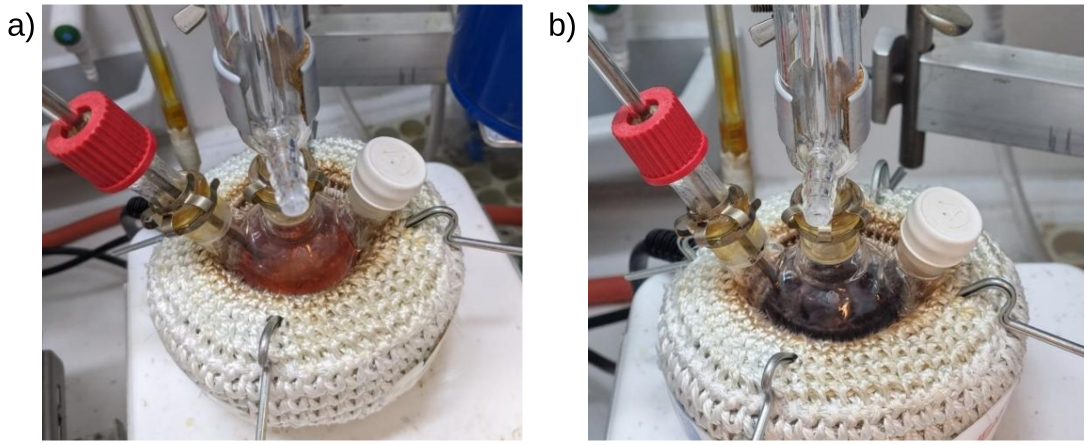

Figure S1: a) Picture of the CuBr-P(^{i}Bu)$_2$H-OLAM reaction solution at 100°C prior to the formation of Cu nanocrystals. b) Picture of the same solution at 200°C after Cu NC formation.

Solution ^{31}P{^{1}H} and ^{1}H nuclear magnetic resonance (NMR) spectroscopy confirm the formation of a molecular complex between CuBr and P(^{i}Bu)$_2$H (**Figure S2**). We observe a downfield shift and broadening of the di-isobutyphosphine ^{31}P resonance (**Figure S2a**). This shift is indicative of an electronic density decrease on the ligand upon coordination to the Cu(I) centers, similarly to what is observed with other phospines.[2,3] This electronic density decrease is also evidenced by ^{1}H NMR (**Figure S2b**). The di-isobutylphospine P-H proton resonances shift downfield and the $^1J_{PH}$ coupling constant (i.e. the distance between the two P-H resonances) increases, as expected for P(^{i}Bu)$_2$H coordination to a metallic center.[7]

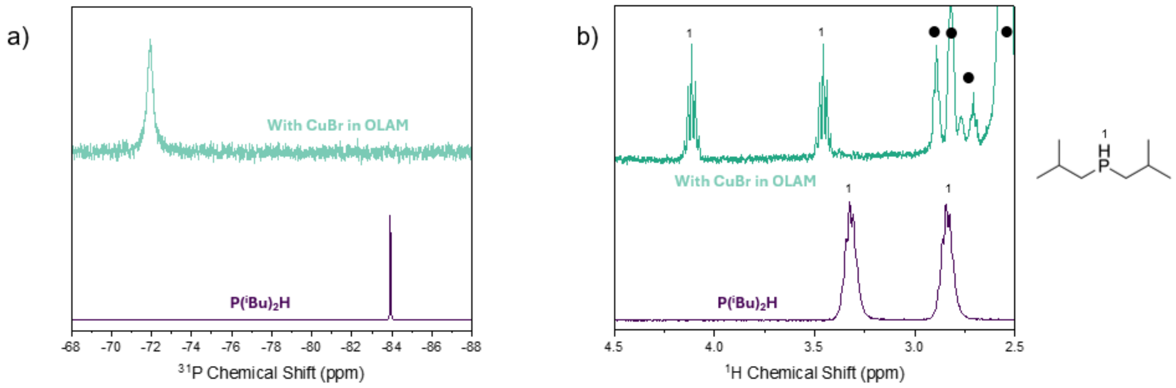

Figure S2: a) ^{31}P{^{1}H} NMR spectrum of an OLAM-CuBr-P(^{i}Bu)$_2$H solution with a 1:1 CuBr:P(^{i}Bu)$_2$H ratio (in green) and the P(^{i}Bu)$_2$H reference spectrum (in purple). (B) ^{1}H NMR spectrum of the same solution (in green) along with the P(^{i}Bu)$_2$H reference spectrum (in purple). The resonances indicated by (●) are attributed to OLAM. Proton 1 appears as a doublet due to P–H coupling. The NMR solvent is toluene-d8.

These results are similar and in line with those that we observed in our previous study with PPh$_2$H.[2] Detailed synthetic and mechanistic insights into the formation of these spherical Cu NCs with di-isobutylphosphine are beyond the scope of this work. However, it is very likely that metallic Cu is also

formed through the disproportionation reaction of the CuBr-P(^{i}Bu)$_2$H molecular complexes, as already evidenced for TOP, TOPO and PPh$_2$H.[2,3] Differences in the reaction thermodynamics or kinetics may account for the different Cu NCs morphologies obtained with P(^{i}Bu)$_2$H (i.e. spheres) and PPh$_2$H (i.e. cubes).

S2 Characterizing the nanocrystals

S2.1 Linear combination fitting of XANES spectra

To extract oxidation states for the as-synthesized Cu-TDPA and Cu-PR_2H nanocrystals, we performed linear combination fitting of the x-ray absorption near edge structure using standard spectra (**Figures S3a-d**). Fitting was performed by testing all possible combinations of Cu foil, Cu_2O and CuO reference spectra to minimize the χ^2 with the experimental spectra (**Figures S3a-f**). The best fits (minimum χ^2) yielded a metallic fraction of 80% for Cu-PR_2H (**Figure S3a**), with the remainder being Cu_2O. Reasonable fits for Cu-PR_2H can also be obtained with only the metallic component, with a reduced χ^2 of $8.0*10^{-4}$ instead of $5.8*10^{-4}$ (**Figure S3a,c**). This is reinforced visually by the overlaid spectra, indicating that the main features of the XANES spectrum of Cu-TDPA are captured by the metallic foil standard (**Figure S3a**). On the contrary, other possible models included only oxidic copper, and lead to a poor recreation of the sample spectrum after least-squares fitting (**Figure S3b,c**). The same models were tested for Cu-TDPA, where the best fit yielded a metallic fraction of 88%, with the remainder once again being best fit by either oxide (**Figure S3d,f**). Leaving out the metallic component fails to reproduce the main features of the XANES spectrum (**Figure S3e,f**).

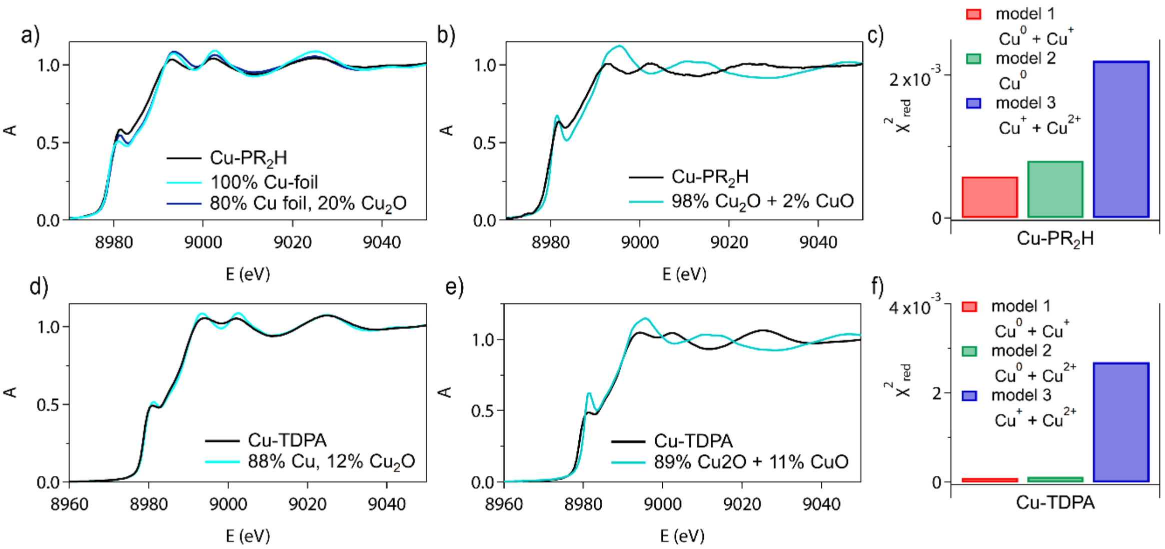

Figure S3: X-ray absorption near-edge spectra (black) and fits (blue) for a,b) Cu-PR_2H and c) reduced χ^2 for the different models. Similar fits and spectra are reported for d,e) Cu-TDPA alongside f) reduced χ^2 of the different models. Percentages are obtained from the coefficients of the linear expression containing standard spectra for Cu foil (Cu^0), Cu_2O and CuO.

S2.2 Diffusion ordered nuclear magnetic resonance spectroscopy (NMR)

Ligand coordination to nanocrystals is identified with the use of solution ^1H NMR. In stable NC dispersions with tightly bound ligands, the protons of the bound ligands are characterized by broadened resonances. Their diffusion coefficient corresponds to the solvodynamic radius of the nanocrystal-ligand complex and can be determined from diffusion ordered NMR spectroscopy (DOSY-NMR).[8,9] NMR spectra of Cu-TDPA and Cu-PR_2H display broadened resonances with a slow diffusion coefficient characteristic of nanocrystal ligation (**Figure S4a,b**). Relative broadening of the proton resonances depends additionally on solvation of the ligand shell and possible exchange equilibria between a bound and unbound state in toluene.[8,9] The sharper resonances of di-isobutylphosphine could arise from a combination of both effects. Indeed, the shorter, branched ligand might be more effectively solvated in toluene compared to TDPA. Additionally, di-isobutylphosphine is a neutral L-type ligands and might be exchanging more rapidly than TDPA, as might be inferred from a slightly faster diffusion coefficient.

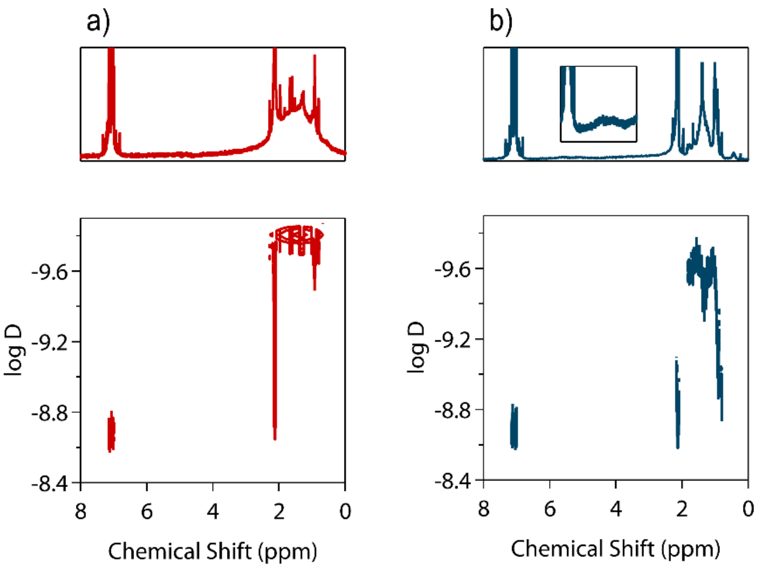

Figure S4: 1D ^1H-NMR spectra and corresponding 2D-DOSY spectra of 2-time purified dispersions of a) Cu-TDPA and b) Cu-PR_2H. The slow diffusing species highlight the bound ligands. Minor contaminations of free species are removed in a 3rd cycle of purification and presented in the main manuscript.

S2.3 Assigning proton resonances in Cu-PR_2H

Reference spectra of oleylamine and di-isobutylphosphine were recorded in toluene and superimposed on the three times purified dispersion of Cu-PR_2H to assign resonances to the respective protons (**Figure S5a,b**). The assignment becomes trivial as the position of the resonances is merely broadened, and residual protons of the oleylamine ($OlNH_2$) backbone can be identified next to dominant resonances of protons in the secondary phosphine.

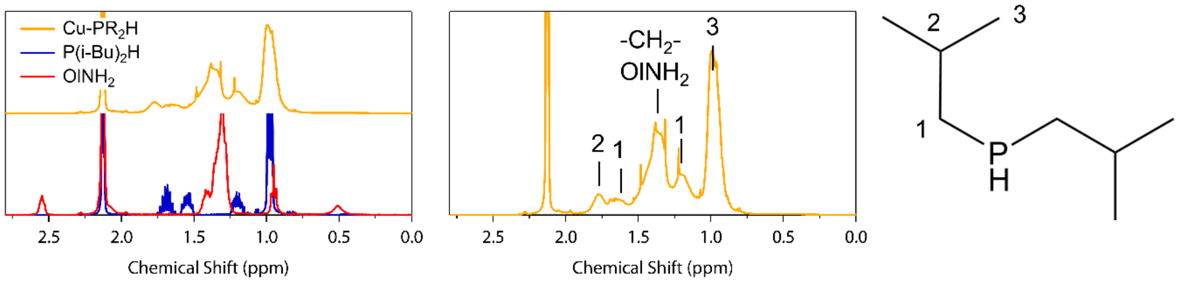

Figure S5: 1D ^{1}H-NMR spectra of di-isobutylphosphine, oleylamine and Cu-PR$_2$H alongside the molecular structure of di-isobutylphosphine. Annotated protons from the structure on the right are indicated on the spectrum.

S2.4 Quantifying the relative composition of ligands on Cu nanocrystals

The nanocrystal surface can be reconstructed and relative ligand amount estimated using a combination of x-ray photoelectron spectroscopy (XPS, **Figure S6**) and quantitative NMR spectra.

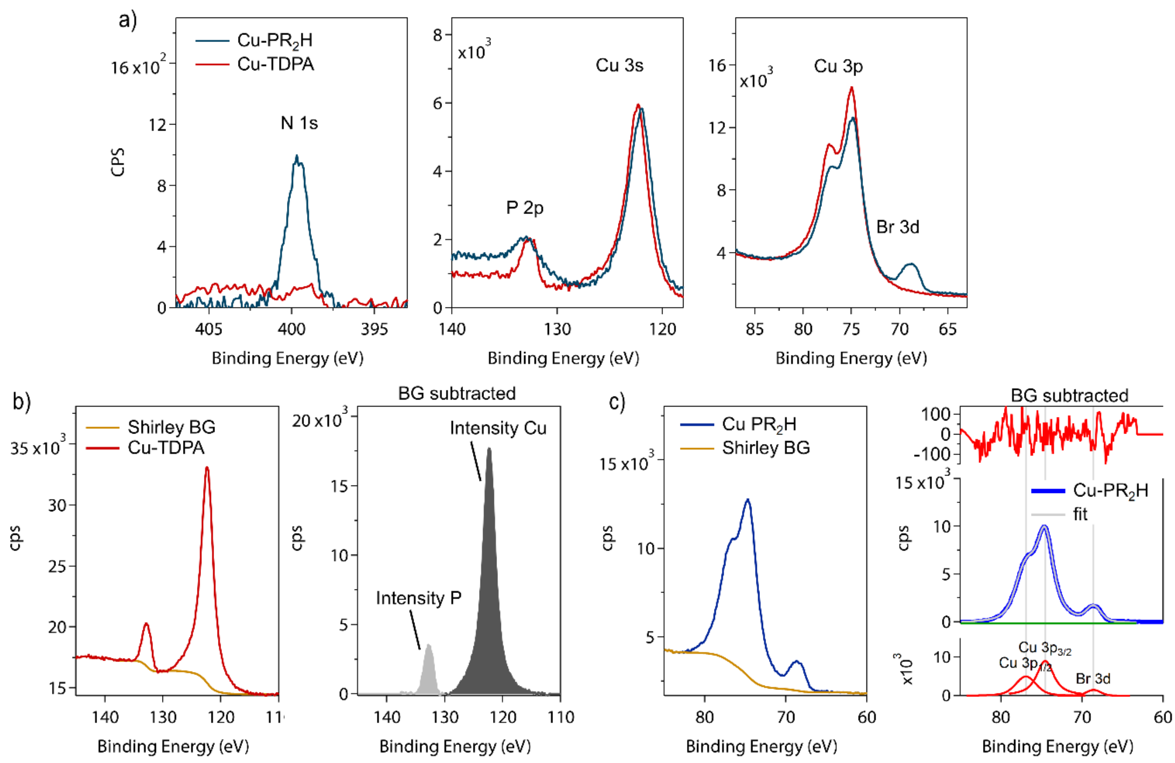

Figure S6: a) X-ray photoelectron spectra at the respective core level binding energies for Cu-TDPA (red line) and Cu-PR_2H (blue line). b) Illustrated procedure to extract the relative intensities for each core-level signal with a Shirley-type background. c) Fitting procedure to extract the Br signal intensity from the background. Relative intensities are then used to calculate the relative composition of the surface with the use of tabulated relative sensitivity factors.

In the case of Cu-TDPA, only trace amounts of N were detected in XPS next to a strong P signal originating from the phosphonate head group (**Figure S6a**). Relative signals of P 2p and N 1s peaks scaled for their respective sensitivity factors are used to estimate surface presence (**Table S1**).[10] The relative peak areas are extracted from a Riemann sum after applying a standard, Shirley-type background correction to the XPS spectrum (**Figure S6b**).[11] A fit is employed in the case of Br in Cu-PR_2H to extract the total signal intensity, due to the overlap between Cu 3p and Br 3d core-levels (**Figure S6c**).The XPS based quantification yields an estimated TDPA surface fraction of 94%. We correspondingly opt to name the sample Cu-TDPA.

	Cu 3p	N 1s	P 2p	Br 3d
Cu-TDPA	1	0.012	0.201	0
Cu-PR_2H	1	0.047	0.207	0.100

Table S1: Estimated relative composition of the samples by X-ray photoelectron spectroscopy. All intensities are relative to the Cu 3p intensity and normalized by their respective sensitivity factors.

For Cu-PR_2H, the ^{1}H-NMR spectrum can be directly used to quantify the relative concentration of bound ligands, and issues from contamination or penetration depth in XPS can be avoided (**Figure S7 a,b**). The NMR spectrum is fitted with 6 peaks, 4 for the respective protons in di-isobutylphosphine and 2 for the aliphatic protons in oleylamine (**Figure S7b**). The area of peak 0, normalized by 12 and 3 for the corresponding methyl protons, and peak 2, normalized by 24 for methylene protons in oleylamine, scale directly with the surface concentration of phosphine and oleylamine according to **Equation 1**. The relative surface concentration of phosphine, calculated as the concentration of phosphine divided by the total ligand concentration is 79 ± 7%. The error estimate is derived from the fit errors. With the relative intensities of P and Br from XPS and their respective relative sensitivity factors (**Table S1**), the Br content is estimated as half of the P content, yielding a relative surface coverage of di-isobutylphosphine, bromine and oleylamine of 1:0.5:0.25. The observation made with commercial Cu upon addition of PR_2H provides strong evidence that the secondary phosphine accounts for the observed behaviour in the Cu-PR_2H while oleylamine and bromide play a marginal role, if any at all. As such, we center the discussion in the main text on PR_2H and name the sample Cu-PR_2H.

$$\frac{I_{peak\ 0}}{I_{peak\ 2}} = R = \frac{3n_{OlNH_2} + 12n_{PR_2H}}{24n_{OlNH_2}}$$ **Equation 1**

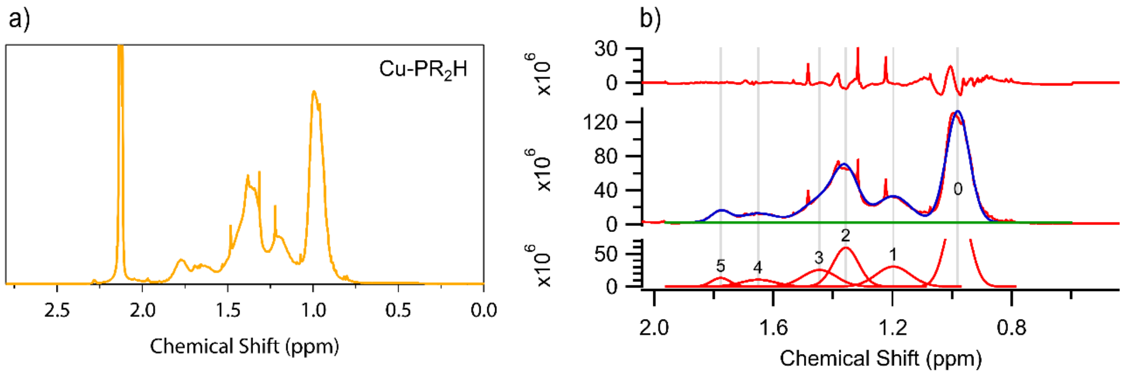

Figure S7: a) 1D ^{1}H-NMR spectrum of Cu-PR_2H. b) Fit results of fitting the aliphatic region of the Cu-PR_2H NMR spectrum with 6 gaussians to estimate the relative concentration of oleylamine and secondary phosphine on the Cu-PR_2H surface.

S3 Additional electrochemistry data

S3.1 Selectivity of nanocrystal catalysts after 1 hour of CO2RR

We measured 1 hour of chronoamperometry with Cu-PR_2H and Cu-TDPA at different potentials to probe eventual differences in product selectivity (**Figure S8**). Interestingly, the product distributions and current densities fall within the error margin at the 1-hour timestamp.

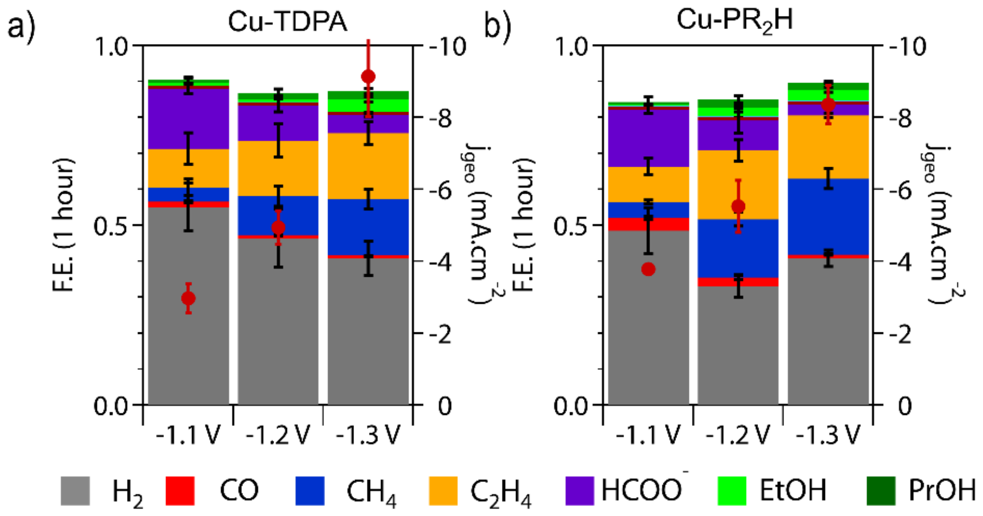

Figure S8: Faradaic efficiency after 1 hour of CO2RR at different potentials with a) Cu-TDPA and b) Cu-PR_2H. Data presented is the average of 3 separate runs. Error bars are the standard deviation across the 3 runs.

S3.2 Faradaic efficiency of gas product generation with Cu nanocrystals

In accordance with the trends in current density presented in the main manuscript, ethylene selectivity shows an immediate maximum in Cu-TDPA, whereas in Cu-PR$_2$H the faradaic efficiency first rises slowly, maintains a maximum for longer, and then drops (**Figure S9, S10**).

The differences among Cu-PR$_2$H and Cu-TDPA in the remaining gas products are more subtle compared to those observed for ethylene, yet noticeable**.** In particular, the hydrogen and methane selectivity and activity decrease on Cu-PR$_2$H when ethylene current density starts to increase (**Figure S9, S10**). This correlation suggests that as ethylene current density picks up, CO-coupling is favored over CO hydrogenation to form methane or HER.

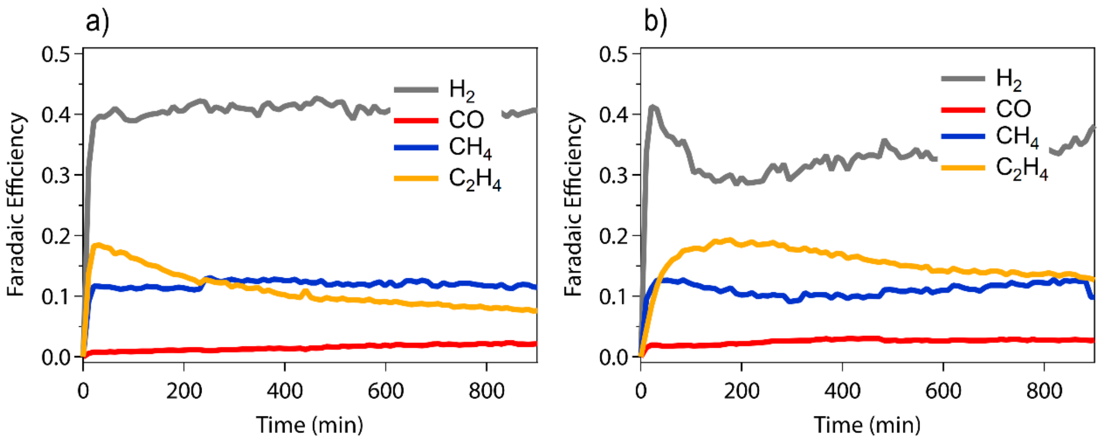

Figure S9: Average faradaic efficiencies of the gas products generated during 15 hours of continuous CO_2RR with a) Cu-TDPA and b) Cu-PR$_2$H. Gas products are sampled every 10 minutes, and the faradaic efficiency is averaged over 4 runs.

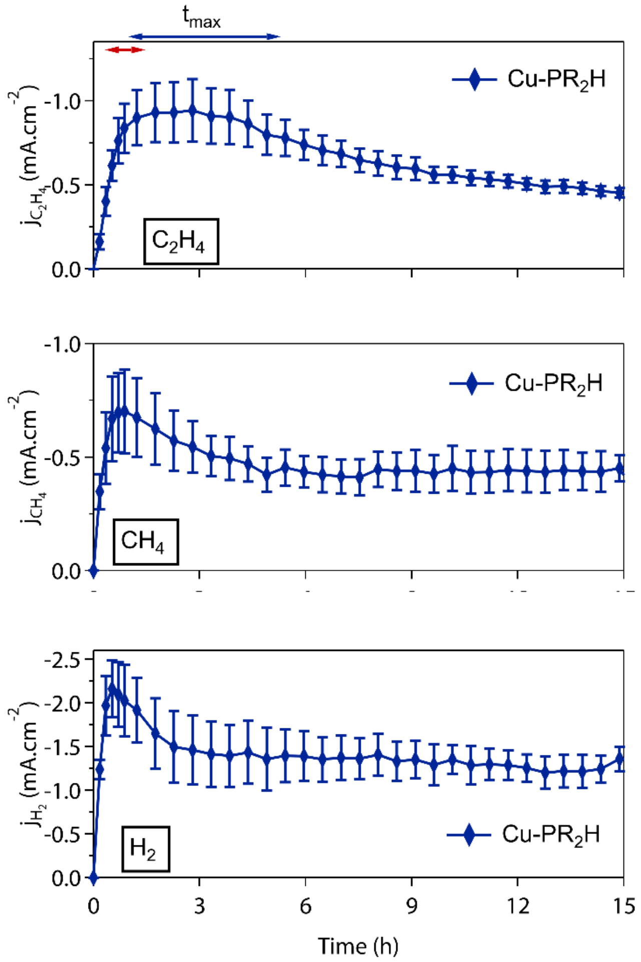

Figure S10: Average current densities of the relevant gas products generated during 15 hours of continuous CO_2RR with Cu-PR_2H. The slow buildup of ethylene is accompanied by a reduction in methane and hydrogen current densities, mirroring the changes in Faradaic efficiency.

S3.3 Selectivity of commercial Cu electrodes modified with Nafion and additives

First, we compared to the ligand free commercial catalyst with the Cu catalysts wherein PR2H was added to the ink (**Figure S11**).

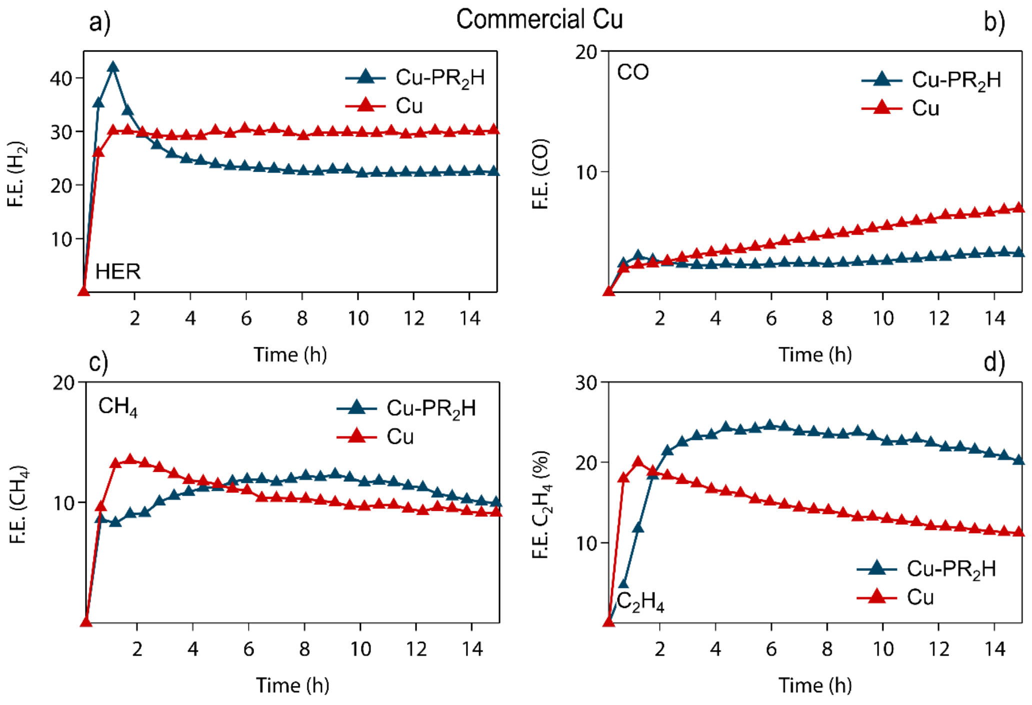

Figure S11: Average faradaic efficiency of the gas products generated during 15 hours of continuous CO_2RR at -1.2 V_{RHE} with commercial Cu catalysts.

Second, we considered the addition of TDPA to the ink (**Figure S12**) which shows that no difference is observed with the ligand free commercial catalyst. The inability of TDPA to alter the stability agrees tion with our model NCs coated with TDPA.

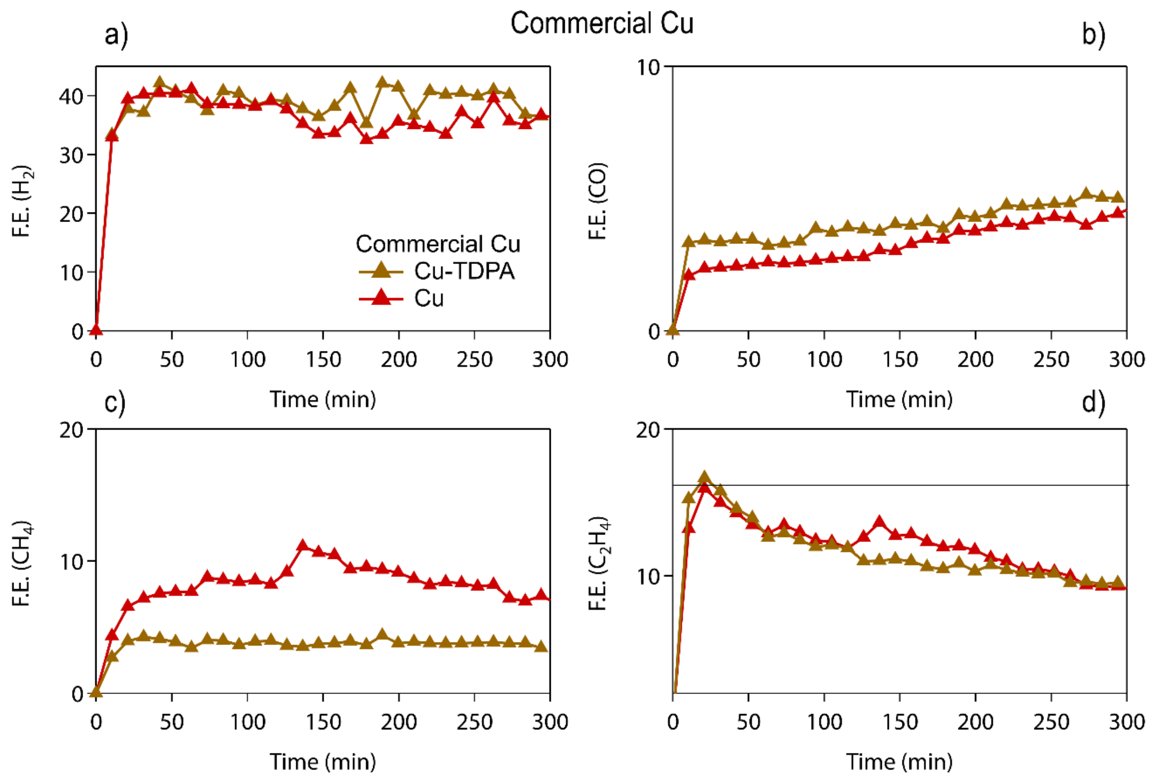

Figure S12: Average faradaic efficiency of the gas products generated during 15 hours of continuous CO_2RR with commercial Cu catalysts (30 µg/cm^2) and 5 mass% Nafion. Faradaic efficiency for a) H_2, b) CO, c) CH_4 and d) C_2H_4.

After reproducing the enhanced ethylene stability of Cu-PR$_2$H NC catalysts by adding 0.5 equivalents of phosphine to commercial Cu, we increased the loading of the ligand further (**Figure 13**). At 2 equivalents, corresponding to a ligand density of 15.4 nm^{-2}, the ethylene activity of commercial Cu is suppressed (**Figure S13**). However, the ethylene current density is similarly stabilized as with 0.5 equivalents, hinting at the same mechanism at the root of operational stability while a balance between initial site passivation and ligand release via applied voltage must be achieved.

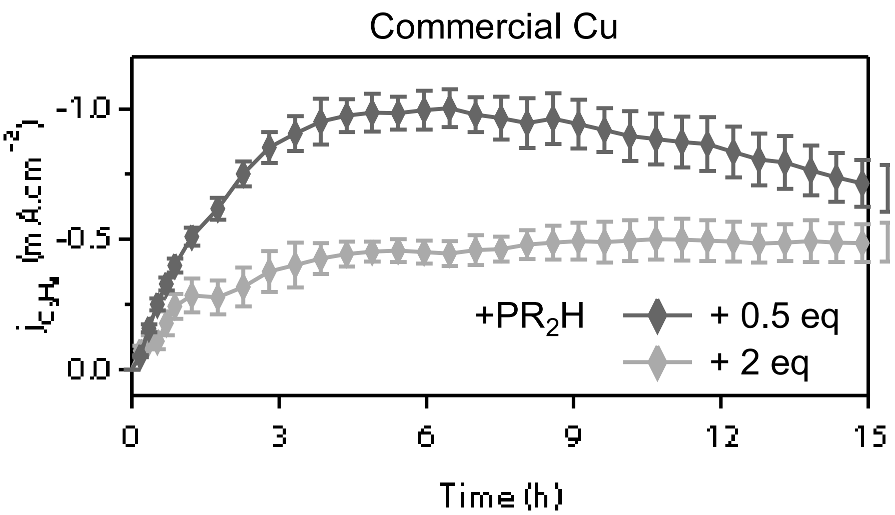

Figure S13: Ethylene specific current density from commercial Cu catalyst electrodes with varying amounts of phosphine ligand in the catalyst ink. The trace of 0.5 eq corresponds to the data from the main manuscript.

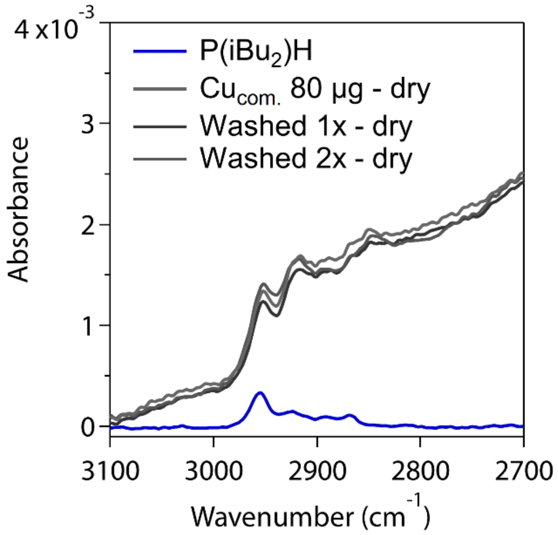

Figure S14: ATR-FTIR spectrum of diisobutylphosphine on commercial Cu. The spectra were collected soon after depositing 80 µg of commercial Cu from a dispersion containing 0.5 mol% diisobutylphosphine (80µg-dry). Subsequently, the electrodes were washed with water and then dried for the measurement (1x-dry and 2x-dry). The persistence of the ligand resonances indicates that the phosphine chemisorbs on the surface of commercial Cu.

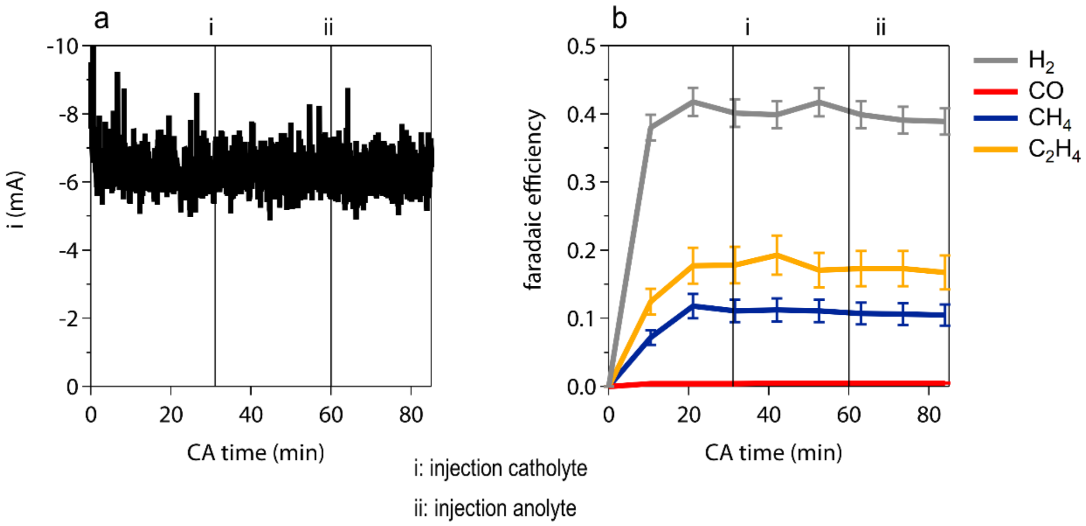

Figure S15: a) Current and b) selectivity of a control experiment where diisobutylphosphine is injected into the catholyte and anolyte during catalysis with a Cu-TDPA NC cathode to yield a concentration of 2.3 micromolar, which is the same amount adsorbed on the NC surface. The negligible changes of the current and selectivity demonstrate that dissolved phosphines in the electrolyte are too dilute to significantly impact CO_2RR at concentrations corresponding to the entire ligand shell desorbing from the NC surface in the assumption that the desorbed ligands fully dissolve in the electrolyte.

S4 ATR-FTIR

S4.1 Benchmarking graphene on Si ATR crystal electrodes

Custom graphene-on-Si ATR crystals were used for the in-situ ATR-FTIR experiments. Graphene was transferred from a Cu foil with the use of a polymethylmethacrylate cover layer, etching of the Cu with ammonium persulfate and direct scooping onto the commercial Si ATR crystals.[12] To determine if the fabricated electrodes were suitable for operando experiments, we electrochemically characterized the graphene and compare voltammetry results of Cu nanocrystals on graphene to Cu nanocrystals on flat glassy carbon electrodes (**Figure S16**). Voltammetry results demonstrate that the same redox transitions and peaks are observed independent of substrate. The increased peak width could arise from a higher resistance of graphene, which is estimated to be between 10 and 20 times higher than on glassy carbon from electrochemical impedance spectroscopy. The lower current is additionally attributed to the lower surface area of the operando electrochemical cell and the increased resistance. Besides the minor differences in the respective voltammograms, the restructuring of Cu is also equivalent on glassy carbon and graphene electrodes. We conclude that the graphene contact layers are a good model system for our glassy carbon substrates to perform operando studies on non-conductive nanocrystal thin films in electrochemical cells.

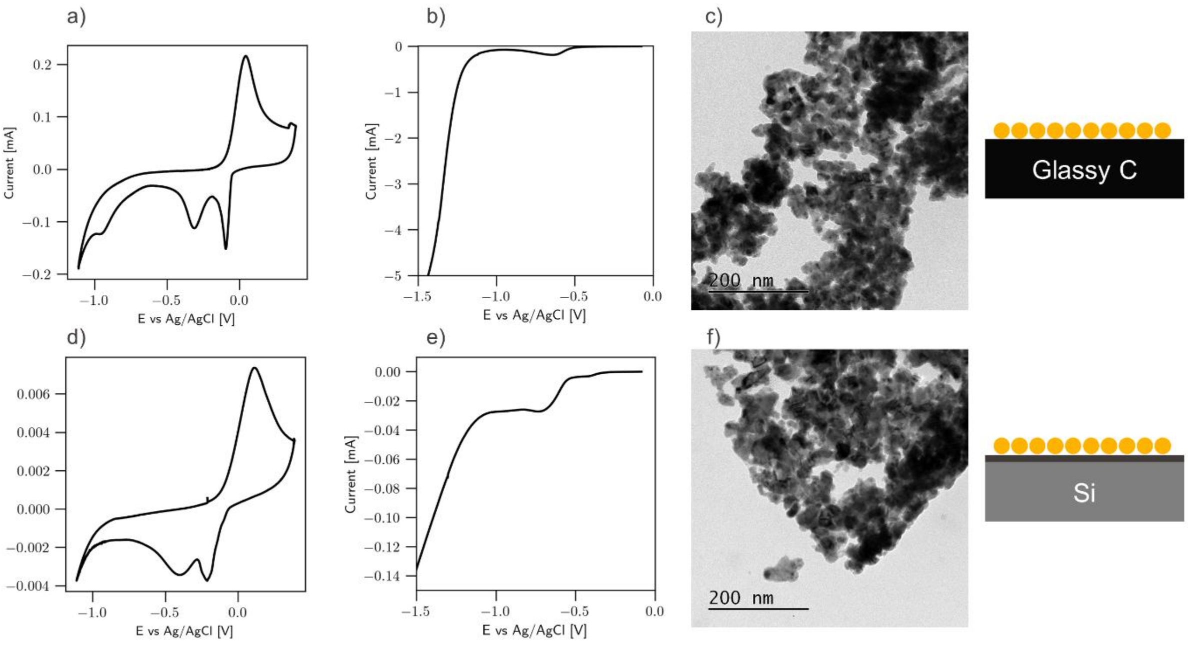

Figure S16 a) Cyclic voltammetry, b) linear sweep voltammetry and c) post-chronoamperometry electron microscopy of Cu-TDPA on glassy carbon. d) Cyclic voltammetry, e) linear sweep voltammetry and f) post-chronoamperometry of Cu-TDPA on graphene.

S4.2 Confirming ligand identification from ^{1}H-NMR results with FTIR

To confirm the presence of both oleylamine and di-isobutylphosphine on the surface of Cu-PR_2H during in-situ FTIR measurements, we recorded the spectra of both molecules and the purified, drop-casted nanocrystal films (**Figure S17**). The C-H stretching vibrations of both molecules show a distinct intensity profile because of the relative intensity of methyl and methylene groups. The IR absorption spectrum of Cu-PR_2H is a superposition of both ligand spectra, and the spectrum can be closely recreated with a linear combination of the phosphine and amine spectra. Quantification of the ligands according to this procedure would be unreliable, as the relative molar extinction coefficients on the surface of Cu are not known and might scale depending on the distance to the metallic nanocrystal.

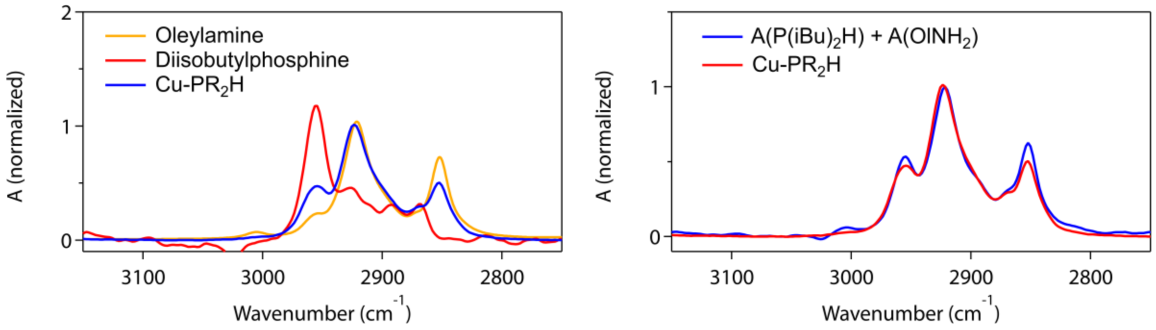

Figure S17: ATR-FTIR spectra of oleylamine, di-isobutylphosphine and Cu-PR_2H (left) and the superposition of both ligand spectra overlaid with the nanocrystal spectrum (right).

S4.3 Quantifying ligand desorption

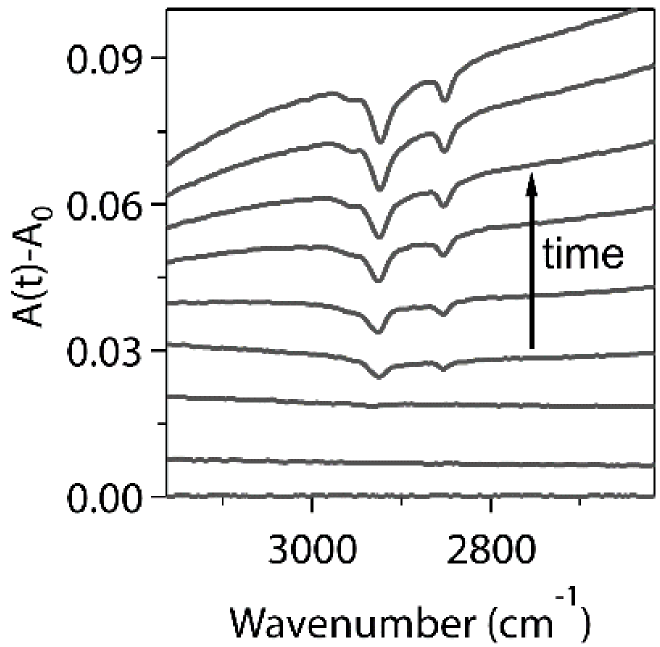

Figure S18: a) Differential ATR-FTIR spectra of Cu-TDPA during chronoamperometry at -1.2 V vs RHE.

To quantify ligand desorption during chronoamperometry, the absorbance of the C-H stretching vibrations was monitored as a function of time. Rather than fitting the absorption spectrum, the difference spectrum with the one acquired at the start of the chronoamperometry was calculated (**Figure S18**). The ensuing negative absorbance (ΔA) was integrated after a polynomial background subtraction, which scales directly to the number of desorbed ligands (**Figure S19**). The absolute ligand density can not be calculated from these absorbances because the absorption coefficient is unknown, so we resort to calculating the relative surface coverages by comparing the desorbed ligand amount to the initial ligand amount. From the integrated negative absorbance at each timestamp, the surface coverage θ is estimated by adding the differential integrated absorbance (ΔA) to the initial integrated absorbance A_0 and normalizing by A_0 (Equation 2):

$$\theta = \frac{A_0 + \Delta A}{A_0} \qquad \text{Equation 2}$$

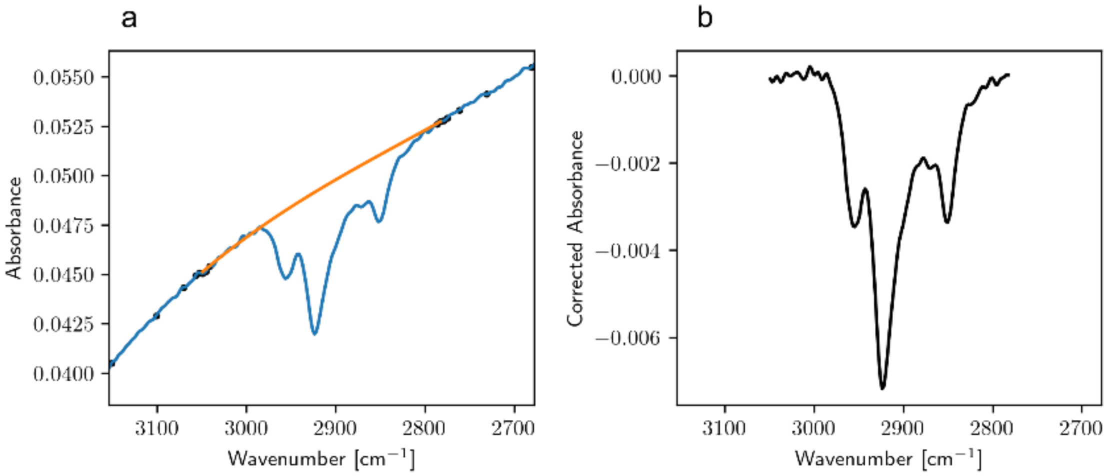

Figure S19: a) Differential ATR-FTIR spectrum of Cu-PR_2H after 1 hour of chronoamperometry with superimposed polynomial background fit. b) Background subtracted differential absorbance spectrum used for integration and to estimate the desorbed ligand fraction.

S4.4 CO adsorption, identifying the onset of CO_2RR

To estimate the onset of CO_2RR, we leveraged the appearance of the bound *CO signal between 1900 and 2100 cm^{-1}. The integrated absorbance of the *CO signal was quantified by performing a polynomial background subtraction followed by a Riemann sum (**Figure S20**). In the main manuscript, this integrated absorbance is tracked to estimate the onset of CO adsorption. The obtained value does not allow for a quantitative interpretation in terms of surface coverage because neither maximum coverage or absorption coefficients are known.

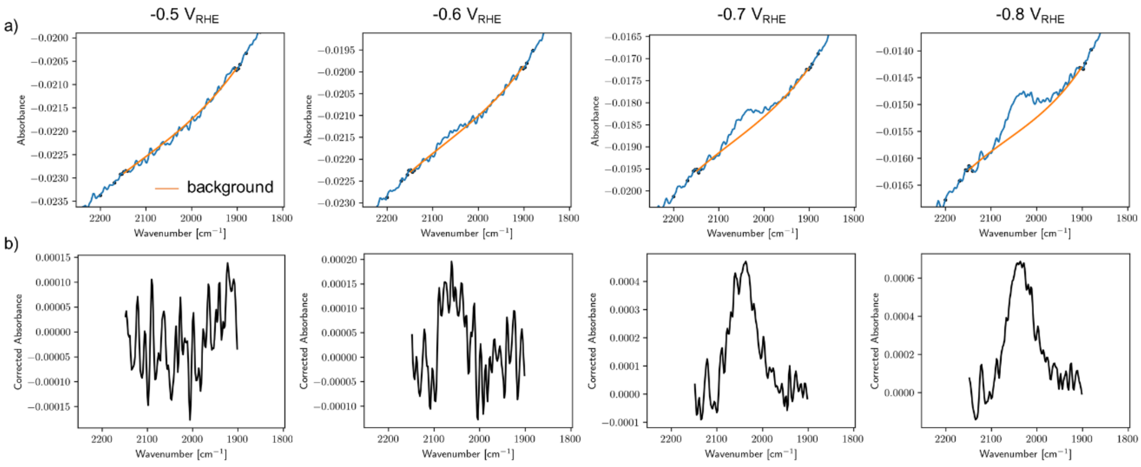

Figure S20: Surface bound *CO signal appears out of the background around -0.6 V vs RHE during a linear sweep voltammogram. *CO surface coverage is correlated to the integrated absorbance following background subtraction. a) Raw spectrum in the wavenumber region around the *CO peak. b) Background subtracted *CO peak.

S4.5 Fitting ligand desorption with two-site kinetics

As discussed in the main manuscript, the integrated absorbance of the C-H resonances is used as an estimate of the ligand surface coverage. We fit the surface coverage θ during chronoamperometry as a function of time with a parallel two-site desorption model with two characteristic time constants τ_1 and τ_2 (**Figure S22**, **Equation 3**). A two-site adsorption model was chosen to accurately fit the desorption data, with precedent in nanocrystal ligand studies.[13]

The final surface coverage θ_f, pre-exponential factors A and B, as well as desorption time constants τ_1 and τ_2 are fit to describe the experimental traces in the main manuscript and are reproduced here (**Table S2**). The dominant desorption process, as determined by the integral $(A_i\tau_i)$, illustrates that ligand desorption in Cu-PR_2H is approximately 10 times slower than in Cu-TDPA.

The two-sites can be different facets, edges, corners, to which the ligands bind with different energies.[13–15] In the case of spherical NCs, one could envision the (111) and (100) facets as possessing different binding energies towards the ligands. However, follow up studies will be needed and would be interesting as controlled ligand desorption from certain sites might be utilized to target the formation of specific grain boundaries in the deriving catalyst.

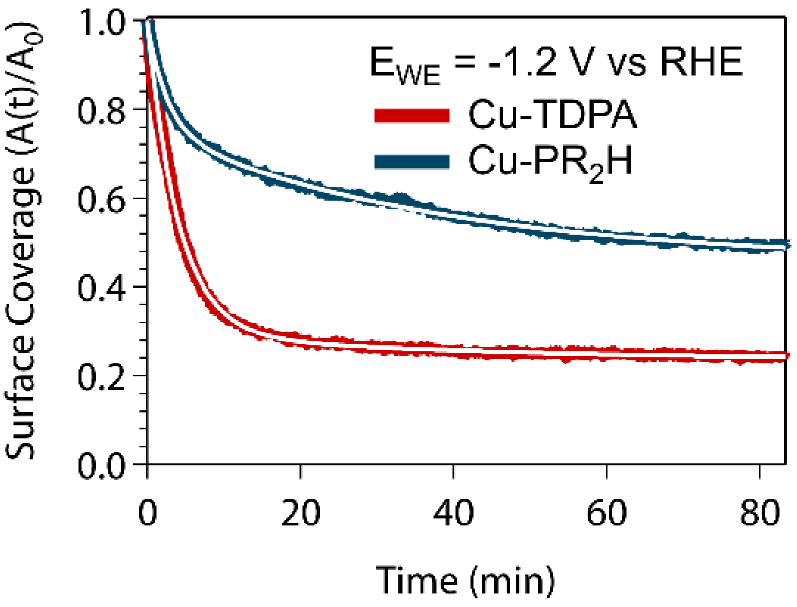

Figure S21: Desorption kinetics presented in the main manuscript. White traces are fit lines obtained with the two-site desorption kinetics described below.

$$\theta(t) = \theta_f + Ae^{-\frac{t}{\tau_1}} + Be^{-\frac{t}{\tau_2}} \quad \text{Equation 3}$$

	θ_f	A	τ_1 (s)	B	τ_2 (s)
Cu-TDPA	0.23	0.70	240	0.06	2319
Cu-PR_2H	0.45	0.23	155	0.30	2265

Table S2: Best-fit parameters for the parallel two-site desorption model to describe ligand desorption from Cu nanocrystals during chronoamperometry. Shaded fields represent the dominant ligand desorption mechanism according to their respective integrals.

S4.6 The desorption lineshape on Cu-PR_2H

The mixed ligand shell of Cu-PR_2H contains primarily diisobutylphosphine and a minor fraction of oleylamine. We investigated whether ligand desorption is selective for either molecule using the in-situ ATR-FTIR (**Figure S21**). The aliphatic backbone of oleylamine and diisobutylphosphine have a different signature of methylene and methyl vibrations. If we isolate the difference spectrum at each point in time during chronoamperometry and compare it to the original spectrum, we actually observe that the difference spectrum has the same relative intensities as the original spectrum, and thus the entire organic ligand shell desorbs at the same time.

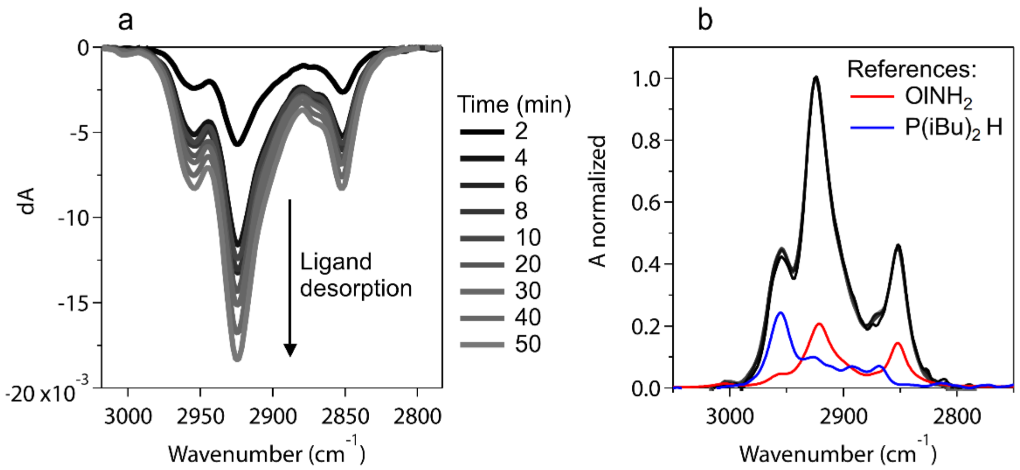

Figure S22: Ligand desorption for Cu-PR_2H during chronoamperometry. a) Overlaid differential absorbance spectra during chronoamperometry performed at -1.2 V_{RHE}. b) Normalized differential absorbance spectra with overlaid pure spectra of oleylamine and isobutylphosphine indicate that both molecules desorb together.

S5 Operando and post-mortem characterization

S5.1 Operando x-ray absorption spectroscopy and linear combination analysis

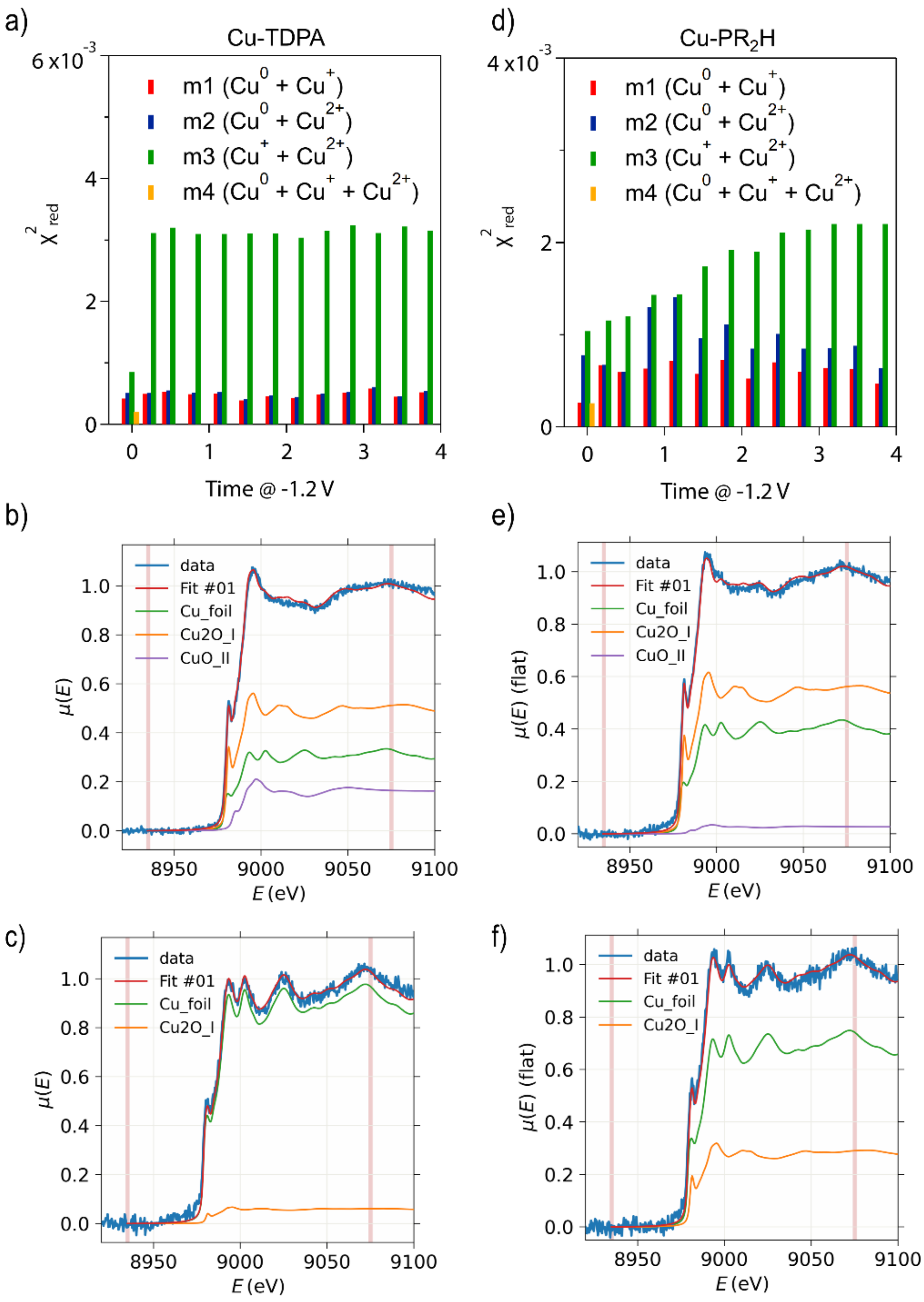

Figure S23: Output of the linear combination analysis of x-ray absorption spectra reported in Figure 3 of the main manuscript. Different models are constructed with reference spectra, and fit to the experimental data with least-squares fitting. The minimum reduced χ^2 at minimum complexity is used to choose the best model. a) Reduced χ^2 for the 4 models as a function of reaction time to fit the evolution of Cu-TDPA speciation during CO_2RR. Best fits for Cu-TDPA spectra at b) open circuit potential and

c) cathodic bias after 3 hours. Equivalent analysis for Cu-PR$_2$H including d) reduced χ^2 for each model as a function of CO_2RR time, e) best fit for the spectrum at open circuit potential as well as f) cathodic bias after 3 hours.

S5.2 Additional post-chronoamperometry electron microscopy

The morphological evolution of Cu-PR$_2$H is slower and more subtle than the instantaneous reconstruction of Cu-TDPA at reducing bias. The structures observed in TEM at different times of continuous chronoamperometry at -1.2 V_{RHE} are reported in **Figure S24**.

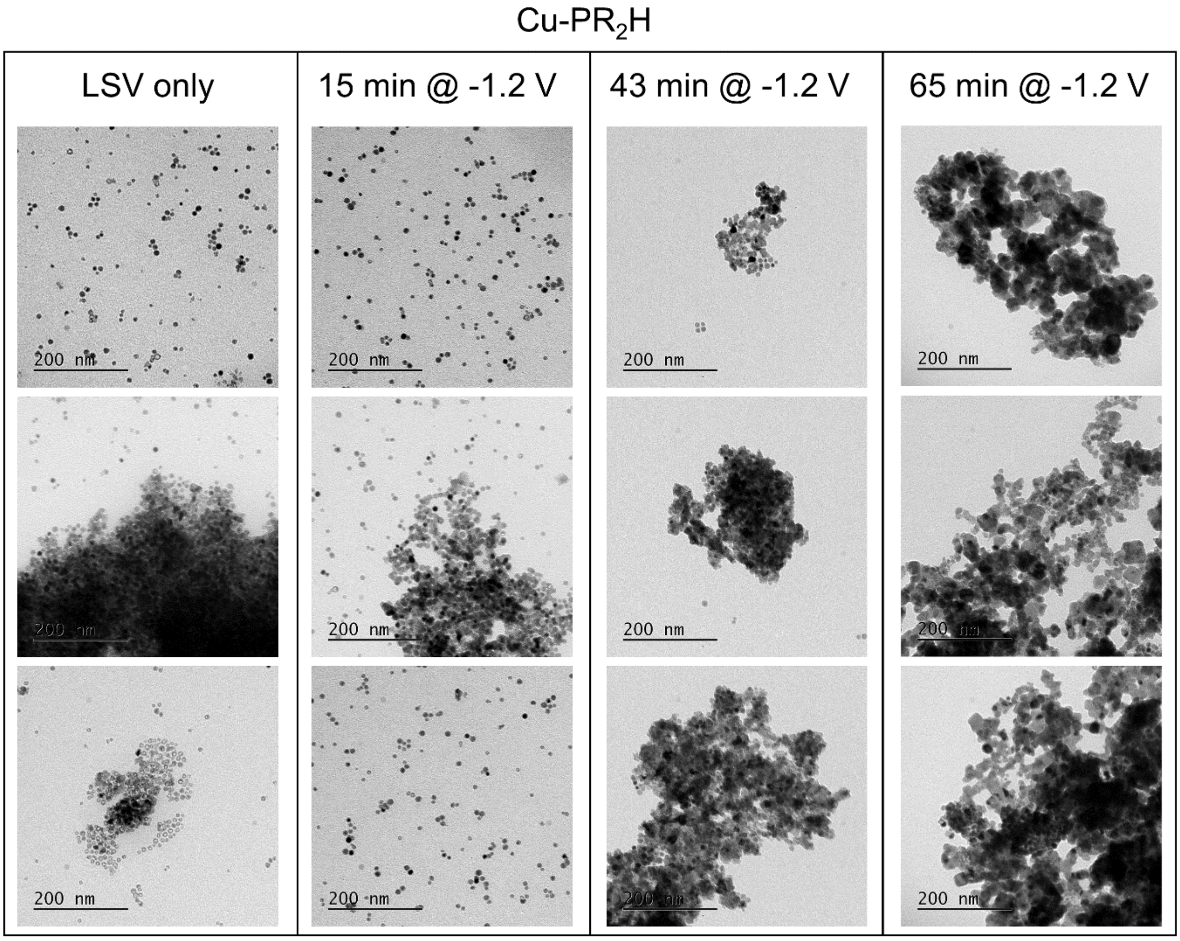

Figure S24: Transmission electron microscopy images of Cu-PR$_2$H at different times under reducing bias. LSV refers to linear sweep voltammetry from open circuit potential to -1.3 V vs RHE. A similar LSV measurement preceded each chronoamperometry measurement.

S6 Simulations to probe ligand effects in CO2RR intermediate adsorption

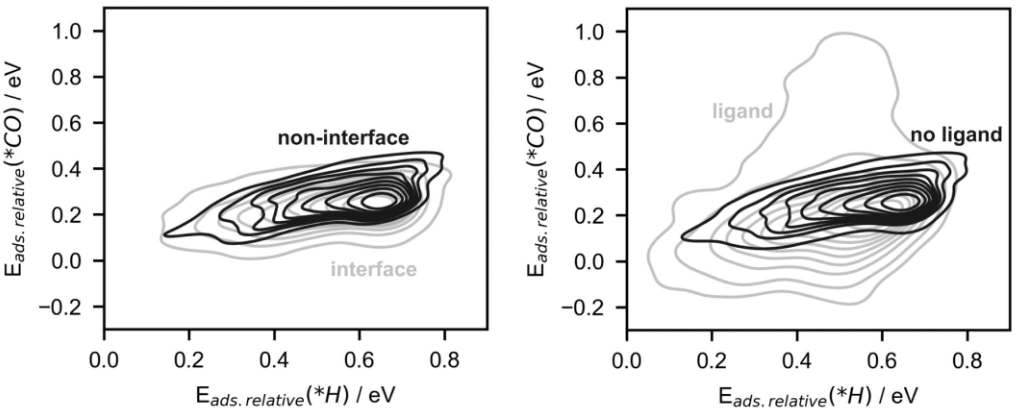

Figure S25: (left) Distribution of adsorption energies for *CO and *H on bare Cu dimer model summarizing binding sites at the interface and the remainder of the model dimer. Less positive adsorption energies for *CO at the interface highlight a potential tendency to promote a local higher *CO surface density. (right) *CO and *H adsorption energies in the presence and absence of ligands reproduced from the main text.

S6.1 Supplementary Discussion

Cu (electro)catalytic systems have been extensively investigated in prior studies[16–18] owing to their unique energetic properties, particularly in the context of CO_2 reduction. These works describe in detail how local structural[19] and electronic effects[20] determine the character of the catalytic interface. Building on this foundation, our focus shifts from local effects and static active sites to a framework that explicitly incorporates: (i) dynamic restructuring phenomena (arising from morphology changes under operating conditions and over time), and (ii) structure-dependent catalytic behavior (where the surface affinity evolves as the atomic configuration of the particle changes).

To achieve this, we leverage the favorable speed/accuracy trade-off of a state-of-the-art machine-learned interatomic potential (see S5.2, Computational Details) enabling large-scale molecular dynamics simulations. Using this MLIP, we construct atomistic models that capture an inclusive ensemble of possible surface-roughening motifs. Specifically, we developed a procedure to generate and evolve a grain boundary between two spherical Cu nanoparticles. Two Cu spherical particles (sizes ranging from ~100 to ~2000 atoms, **Figure S26**) were brought into contact such that a single Cu-Cu covalent bond was formed, followed by full ionic relaxation. The relaxed structures were then propagated at 300 K by molecular dynamics (MD, **Figure S27**). The procedure was repeated 5 times to ensure repeatability.

To obtain a comprehensive distribution of local affinity towards distinct substrates, we generated a surface manifold as described by a grid "shrinkwrap" procedure.[21] This method ensures strict geometric control and consistency over the manifold vertices, where each vertex is positioned at uniform distance to the nearby Cu sites. Using outer vertex normals, molecular fragments (*H, *CO) were placed and the energy of the total system was evaluated. This procedure allows us to generate a distribution of

chemical affinities as a function of surface position over the whole particle. In addition, following this approach ensured the presence of an “internal reference” within each atomistic model, as non-perturbed, quasi-spherical sites can be considered under identical conditions.

Phosphine ligands were subsequently added (conformer sampled, and distributed on the particles ensuring the correct orientation of the P-Cu bond) using *AutoAdsorbate* heuristics[21], corresponding to the experimentally determined coverage at early timescales when the CO_2RR product distribution starts to shift (θ = 0.6). The Cu atom positions were taken directly from the MD snapshots, while the appended ligands were relaxed to ensure physically meaningful interactions with the Cu particles. The entire initialization procedure was repeated 15 times with different random seeds (**Figure S28**). Following this workflow, we established a platform that enables direct comparison of the effects of surface roughness and ligand presence on an identical ensemble of thermalized active sites, using the internal energy reference provided by the embedded spherical regions. This allows us to compare identical adsorbate geometries in the presence and absence of the ligand (**Figure S29**). Similar to what is expect from the adsorption of *CO at grain boundaries, we identify more negative adsorption energies for *CO at the interface between two particles (**Figure S30**). The lower, and thus more beneficial, adsorption energy for *CO at the grain boundary coincides with the grain boundaries being active sites for ethylene.

S6.2 Computational Details

All calculations were carried out using the Atomistic Simulation Environment suite. Structural relaxations employed BFGS gradient descent, and molecular dynamics simulations were conducted using a Langevin thermostat (300 K, up to 0.5 ns , 0.1 fs^{-1}). Using this approach we demonstrated stable dynamics and observed the evolution of the interface (**Figure S26**, by counting the number of Cu atoms stemming from sphere 1 in interaction with at least one Cu atom of sphere 2). The simulation were driven by the state-of-the-art version of the MACE foundational potential (MLIP, MACE [22]) trained on the comprehensive OMat dataset.[23]

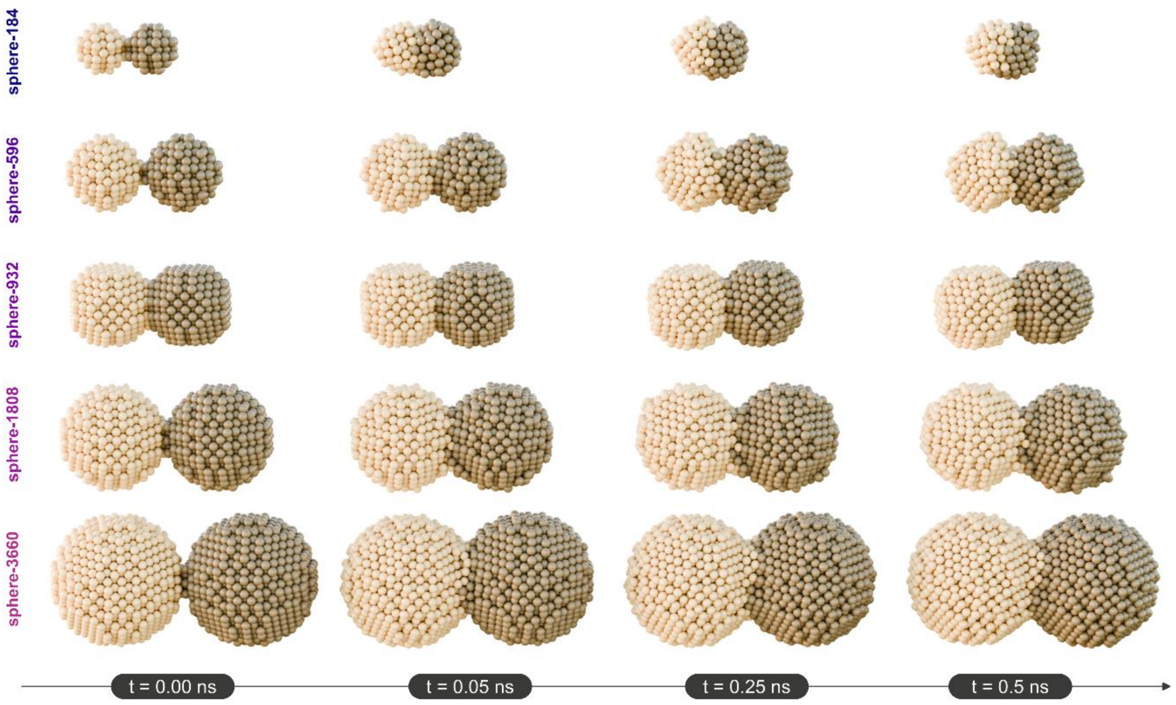

Figure S26 Atomistic representation of molecular dynamics (MD) frames collected during the evolution of a Cu_{sphere}/Cu_{sphere} interface (1 out of 5 repeated MD runs is visualized). Two independently constructed spherical Cu particles (sizes ranging from ~100 to ~2000 atoms) are brought into contact such that a single Cu–Cu bond forms at the interface. The resulting dimer is then subjected to ionic relaxation (t=0 ns), followed by finite-temperature molecular dynamics to induce realistic surface evolution and grain-boundary reconstruction.

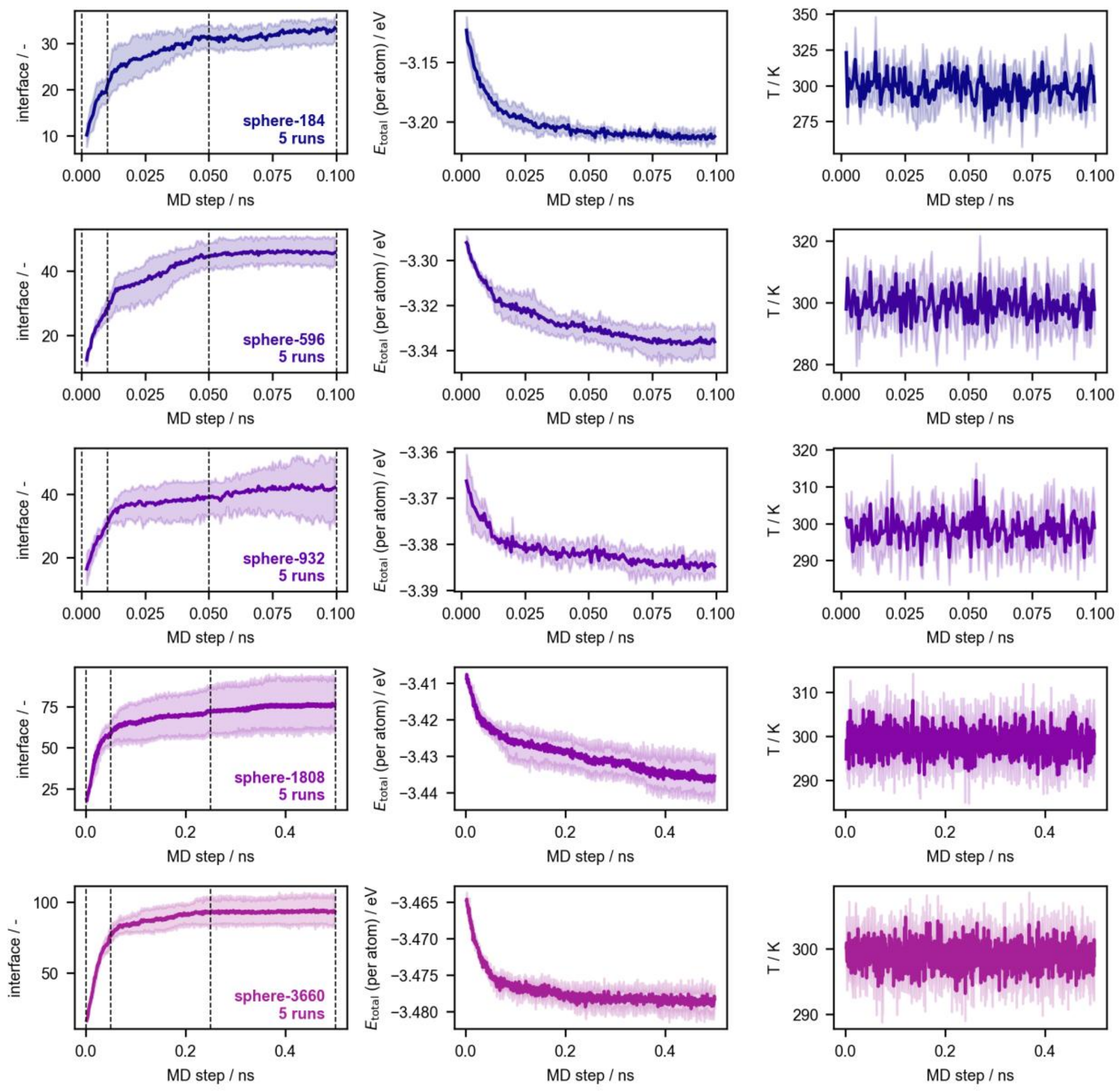

Figure S27 Time evolution of structural and thermodynamic descriptors during MD trajectories for Cu nanoparticles of varying sizes (sphere-184, sphere-262, sphere-596, sphere-932, sphere-1808, sphere-3660). For each system, three quantities are shown as a function of MD step (500 fs per step): (**left**) number of interface interactions formed at the Cu/Cu grain boundary (counted as number of atoms with at least one atom in contact with adjacent particle), (**middle**) total energy per atom, and (**right**) instantaneous temperature. Each row corresponds to one nanoparticle system, with colors consistently assigned across panels. The annotated labels in the left column indicate the system identity.

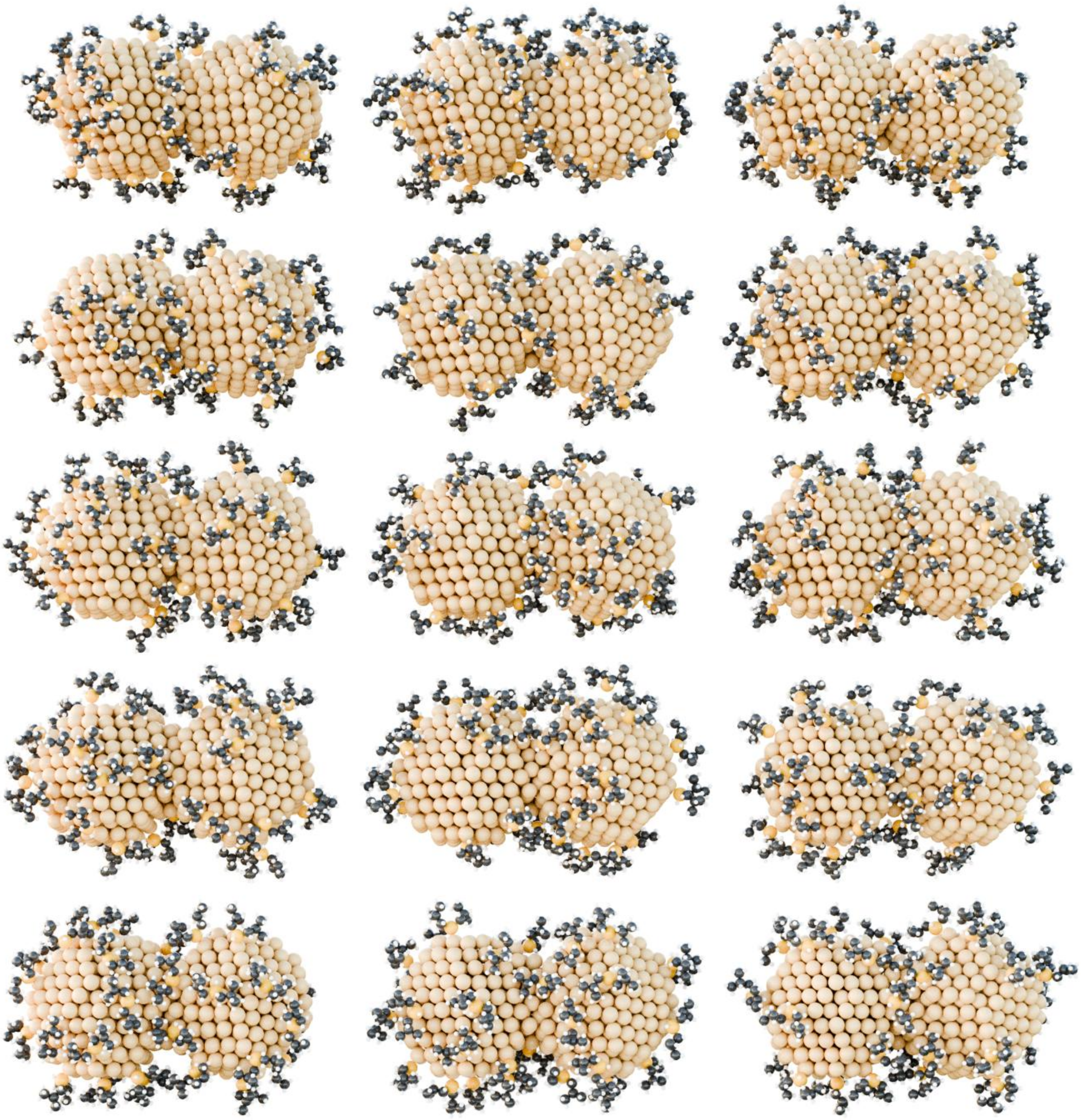

Figure S28 Phosphine ligand covered Cu sphere/sphere interfaces generated by the procedure described in the Supplementary Discussion.

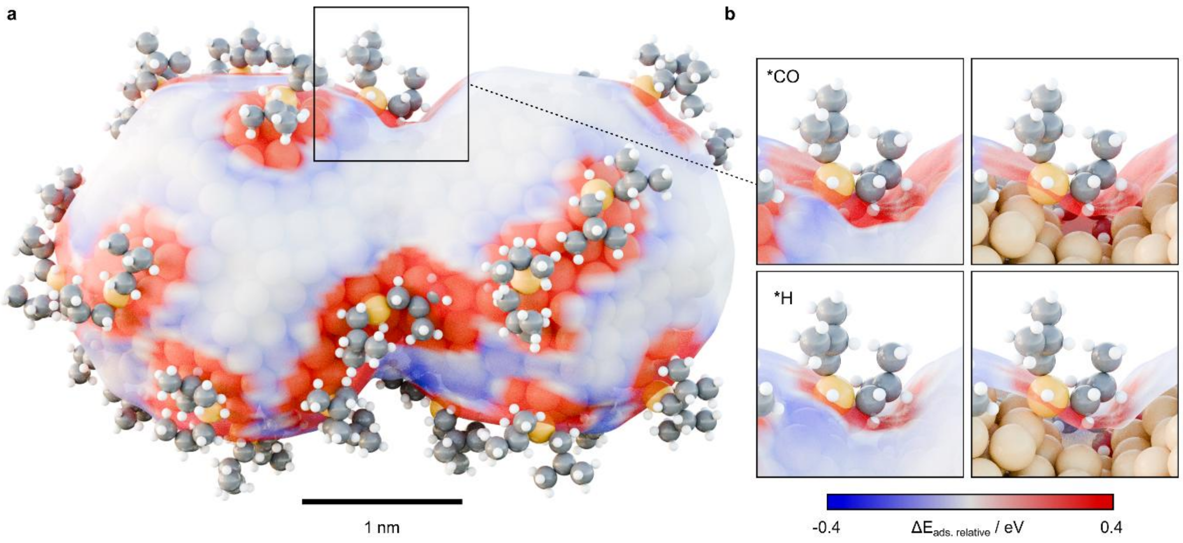

Figure S29 Comparison of the adsorption energy distribution of *CO with and without the presence of phosphine ligands over the surface of a Cu sphere/sphere interface (**a**). The adsorption energy of *CO is calculated for two cases: (i) in the absence of phosphine ligands: $E_{ads,\ no\ ligand} = E_{Cu} + E_{CO} - E_{Cu+CO}$ and in the presence of the ligand: $E_{ads,\ ligand} = E_{Cu+ligand} + E_{CO} - E_{Cu+ligand+CO}$. Comparing these two energies, we can obtain a relative shift in binding affinity as a consequence of the presence of the ligand: $\Delta E_{ads.relative} = E_{ads,\ no\ ligand} - E_{ads,\ ligand}$. Here we can see the areas that are effectively sterically blocked by the presence of the ligand in red, while areas where affinity is increased appears as blue. (**b**) Illustration of the local area affected by the ligand for *CO and *H, showing the underlying geometry of the site. Notice the greater red "zone" for *CO in comparison to *H.

References

(1) Zaza, L.; Ranković, B.; Schwaller, P.; Buonsanti, R. A Holistic Data-Driven Approach to Synthesis Predictions of Colloidal Nanocrystal Shapes. *J. Am. Chem. Soc.* **2025**, *147*, 6116–6125. https://doi.org/10.1021/jacs.4c17283.

(2) Zaza, L.; Stoian, D. C.; Bussell, N.; Albertini, P. P.; Boulanger, C.; Leemans, J.; Kumar, K.; Loiudice, A.; Buonsanti, R. Increasing Precursor Reactivity Enables Continuous Tunability of Copper Nanocrystals from Single-Crystalline to Twinned and Stacking Fault-Lined. *J. Am. Chem. Soc.* **2024**. https://doi.org/10.1021/jacs.4c12905.

(3) Strach, M.; Mantella, V.; Pankhurst, J. R.; Iyengar, P.; Loiudice, A.; Das, S.; Corminboeuf, C.; Van Beek, W.; Buonsanti, R. Insights into Reaction Intermediates to Predict Synthetic Pathways for Shape-Controlled Metal Nanocrystals. *J. Am. Chem. Soc.* **2019**, *141*, 16312–16322. https://doi.org/10.1021/jacs.9b06267.

(4) Suen, N. T.; Kong, Z. R.; Hsu, C. S.; Chen, H. C.; Tung, C. W.; Lu, Y. R.; Dong, C. L.; Shen, C. C.; Chung, J. C.; Chen, H. M. Morphology Manipulation of Copper Nanocrystals and Product Selectivity in the Electrocatalytic Reduction of Carbon Dioxide. *ACS Catal.* **2019**, *9*, 5217–5222. https://doi.org/10.1021/acscatal.9b00790.

(5) Lu, S. C.; Hsiao, M. C.; Yorulmaz, M.; Wang, L. Y.; Yang, P. Y.; Link, S.; Chang, W. S.; Tuan, H. Y. Single-Crystalline Copper Nano-Octahedra. *Chem. Mater.* **2015**, *27*, 8187–8188. https://doi.org/10.1021/acs.chemmater.5b03519.

(6) Guo, H.; Chen, Y.; Cortie, M. B.; Liu, X.; Xie, Q.; Wang, X.; Peng, D. L. Shape-Selective Formation of Monodisperse Copper Nanospheres and Nanocubes via Disproportionation Reaction Route and Their Optical Properties. *J. Phys. Chem. C* **2014**, *118*, 9801–9808. https://doi.org/10.1021/jp5014187.

(7) Phosphorus-31 NMR Spectroscopy; Kühl, O., Ed.; Springer Berlin Heidelberg: Berlin, Heidelberg, 2009. https://doi.org/10.1007/978-3-540-79118-8.

(8) Hens, Z.; Martins, J. C. A Solution NMR Toolbox for Characterizing the Surface Chemistry of Colloidal Nanocrystals. *Chem. Mater*. **2013**, *25*, 1211-1221. https://doi.org/10.1021/cm303361s.

(9) De Roo, J.; Yazdani, N.; Drijvers, E.; Lauria, A.; Maes, J.; Owen, J. S.; Van Driessche, I.; Niederberger, M.; Wood, V.; Martins, J. C.; Infante, I.; Hens, Z. Probing Solvent-Ligand Interactions in Colloidal Nanocrystals by the NMR Line Broadening. *Chem. Mater.* **2018**, *30*, 5485–5492. https://doi.org/10.1021/acs.chemmater.8b02523.

(10) Ward, R. J.; Wood, B. J. A Comparison of Experimental and Theoretically Derived Sensitivity Factors for XPS. *Surface and Interface Analysis* **1992**, *18*, 679–684. https://doi.org/10.1002/sia.740180908.

(11) Oku, M.; Suzuki, S.; Ohtsu, N.; Shishido, T.; Wagatsuma, K. Comparison of Intrinsic Zero-Energy Loss and Shirley-Type Background Corrected Profiles of XPS Spectra for Quantitative Surface Analysis: Study of Cr, Mn and Fe Oxides. *Appl. Surf. Sci.* **2008**, *254*, 5141–5148. https://doi.org/10.1016/j.apsusc.2008.02.003.

(12) Toleukhanova, S.; Shen, T. H.; Chang, C.; Swathilakshmi, S.; Bottinelli Montandon, T.; Tileli, V. Graphene Electrode for Studying CO2 Electroreduction Nanocatalysts under Realistic Conditions in Microcells. *Adv. Mater.* **2024**, *36,* 2311133. https://doi.org/10.1002/adma.202311133.

(13) Drijvers, E.; De Roo, J.; Martins, J. C.; Infante, I.; Hens, Z. Ligand Displacement Exposes Binding Site Heterogeneity on CdSe Nanocrystal Surfaces. *Chem. Mater.* **2018**, *30*, 1178–1186. https://doi.org/10.1021/acs.chemmater.7b05362.

(14) Singh, S.; Tomar, R.; Ten Brinck, S.; De Roo, J.; Geiregat, P.; Martins, J. C.; Infante, I.; Hens, Z. Colloidal CdSe Nanoplatelets, A Model for Surface Chemistry/Optoelectronic Property Relations in Semiconductor Nanocrystals. *J. Am. Chem. Soc.* **2018**, *140*, 13292–13300. https://doi.org/10.1021/jacs.8b07566.

(15) Leemans, J.; Singh, S.; Li, C.; Ten Brinck, S.; Bals, S.; Infante, I.; Moreels, I.; Hens, Z. Near-Edge Ligand Stripping and Robust Radiative Exciton Recombination in CdSe/CdS Core/Crown Nanoplatelets. *J. Phys. Chem. Lett.* **2020**, *11*, 3339–3344. https://doi.org/10.1021/acs.jpclett.0c00870.

(16) Vavra, J.; Ramona, G. P. L.; Dattila, F.; Kormányos, A.; Priamushko, T.; Albertini, P. P.; Loiudice, A.; Cherevko, S.; Lopéz, N.; Buonsanti, R. Solution-Based Cu+ Transient Species Mediate the Reconstruction of Copper Electrocatalysts for CO2 Reduction. *Nat. Catal.* **2024**, *7*, 89–97. https://doi.org/10.1038/s41929-023-01070-8.

(17) Heenen, H. H.; Gauthier, J. A.; Kristoffersen, H. H.; Ludwig, T.; Chan, K. Solvation at Metal/Water Interfaces: An *Ab Initio* Molecular Dynamics Benchmark of Common Computational Approaches. *J. Chem. Phys.* **2020**, *152*. https://doi.org/10.1063/1.5144912.

(18) Cheng, T.; Xiao, H.; Goddard, W. A. Full Atomistic Reaction Mechanism with Kinetics for CO Reduction on Cu(100) from Ab Initio Molecular Dynamics Free-Energy Calculations at 298 K. *Proc. Natl. Acad. Sci. U. S. A.* **2017**, *114,* 1795–1800. https://doi.org/10.1073/pnas.1612106114.

(19) Erhard, L. C.; Schörghuber, J.; Comas-Vives, A.; Madsen, G. K. H. How Realistic Are Idealized Copper Surfaces? A Machine Learning Study of Rough Copper-Water Interfaces**.** *ACS Materials Au* **2025**.

(20) Monteiro, M. C. O.; Dattila, F.; Hagedoorn, B.; García-Muelas, R.; López, N.; Koper, M. T. M. Absence of CO2 Electroreduction on Copper, Gold and Silver Electrodes without Metal Cations in Solution. *Nat. Catal.* **2021**, *4*, 654–662. https://doi.org/10.1038/s41929-021-00655-5.

(21) Fako, E.; De, S. Simple Heuristics for Advanced Sampling of Reactive Species on Surfaces. April 17, 2025. *arXiv preprint* https://doi.org/10.26434/chemrxiv-2025-79nj4.

(22) Batatia, I.; Lin, C.; Hart, J.; Kasoar, E.; Elena, A. M.; Norwood, S. W.; Wolf, T.; Csányi, G. Cross Learning between Electronic Structure Theories for Unifying Molecular, Surface, and Inorganic Crystal Foundation Force Fields. *arXiv preprint arXiv:2510.25380* **2025**.

(23) Barroso-Luque, L.; Shuaibi, M.; Fu, X.; Wood, B. M.; Dzamba, M.; Gao, M.; Rizvi, A.; Zitnick, C. L.; Ulissi, Z. W. *arXiv preprint arXiv:2410.12771* **2024**
